# Spliceosome inhibition induces Z-RNA and ZBP1-driven cell death in small cell lung cancer

**DOI:** 10.1016/j.celrep.2025.116384

**Published:** 2025-10-04

**Authors:** Xinpei Jiang, Xueying Ma, Yunyun Zhou, Xiaodan Liu, Ting Zhang, William Kim, Siddharth Balachandran, Israel Cañadas

**Affiliations:** 1 Nuclear Dynamics and Cancer Program, Fox Chase Cancer Center, Philadelphia, PA, USA; 2 Cancer Epigenetics Institute, Fox Change Cancer Center, Philadelphia, PA, USA; 3 Center for Immunology, Fox Chase Cancer Center, Philadelphia, PA, USA; 4 Biomedical Science Graduate Program, Lewis Katz School of Medicine at Temple University, Philadelphia, PA, USA; 5 Cancer Signaling and Microenvironment Program, Fox Chase Cancer Center, Philadelphia, PA, USA; 6 Laboratory of Hepatic AI Translation, Frontiers Science Center for Disease-Related Molecular Network, West China Hospital, Sichuan University, Chengdu, China; 7 Department of General Surgery, West China Hospital, Sichuan University, Chengdu, China; 8 Liver Transplant Center, Transplant Center, West China Hospital, Sichuan University, Chengdu, China; 9 Moores Cancer Center, UC San Diego, La Jolla, CA, USA; 10 Center for Novel Therapeutics, UC San Diego, La Jolla, CA, USA; 11 Department of Medicine, UC San Diego, La Jolla, CA, USA; 12 Lead contact

## Abstract

Spliceosome inhibitors emerged as promising anticancer agents. Recent studies have demonstrated that spliceosome-targeted therapies (STTs) trigger antitumor immune responses by inducing the accumulation of right-handed double-stranded (ds)RNA (A-RNA), resulting in the activation of RIG-I-like receptors (RLRs) and type I interferon-driven antiviral responses. Here, we show that spliceosome inhibition by pharmacological or genetic neutralization of SF3B1 activity induces the accumulation of endogenous left-handed dsRNAs (Z-RNAs) derived from intron-retained RNAs. These Z-RNAs activate the Z-form nucleic acid-sensor ZBP1, which triggers cell death in mouse embryonic fibroblasts and small cell lung cancer (SCLC) cells. Spliceosome inhibition induced potent ZBP1-dependent cell death in cancer-associated fibroblasts, which was essential for enhancing immunotherapy response in mouse models of SCLC. Collectively, these results demonstrate that spliceosome inhibitors can be used to generate Z-RNA and trigger on-demand ZBP1-dependent cell death in cells of the tumor microenvironment (TME) as a therapeutic strategy to enhance immunotherapy responses in resistant cancers.

## In brief

RNA splicing is frequently mutated in cancer and contributes to its pathogenesis. Jiang et al. demonstrate that spliceosome perturbation induces Z-RNAs, triggering ZBP1-dependent cell death in SCLC cells and CAFs and enhancing immunotherapy responses. Jiang et al. propose STTs as a strategy for therapeutic activation of ZBP1-induced necroptosis in cancer.

## Graphical Abstract

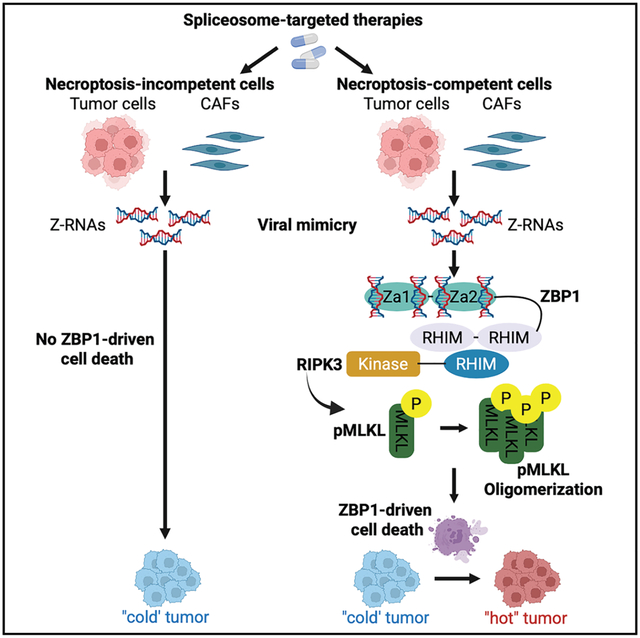

## INTRODUCTION

Spliceosome deregulation, often caused during oncogenesis by perturbation or mutation of RNA splicing factors, is common in cancer.^1–15^ As a result, cancer cells become dependent on aberrant splicing programs that support oncogenic transformation and survival, making them particularly vulnerable to further spliceosome inhibition and positioning small-molecule spliceosome inhibitors as promising antitumor agents.^3,8,16–18^ Interestingly, spliceosome targeted-therapies (STTs) can also trigger intratumoral innate immune responses through the accumulation of dsRNA and the activation of host dsRNA pattern recognition sensors.^18^ The induction of these “virus-mimetic” nucleic acids in cancer cells represents a promising strategy to enhance immune-checkpoint blockade (ICB) responsiveness in immunologically “cold” tumors.^19–25^ Many viral mimicry-inducing approaches in cancer therapy involve the aberrant production of endogenous sources of dsRNAs, which typically adopt the right-handed (A-RNA) conformation and are recognized by A-RNA sensors such as MDA-5 (melanoma differentiation-associated protein 5) and PKR (protein kinase R).^20,26–33^ However, endogenous dsRNAs can also adopt a left-handed conformation (Z-RNAs), which are recognized by the host sensor ZBP1 to trigger a hyper-inflammatory form of nuclear necroptosis during influenza A virus infections.^34,35^ Indeed, we have recently shown that therapeutic activation of ZBP1-driven necroptosis is an effective viral mimicry-inducing means of boosting cancer immunotherapy responsiveness.^36^ While STTs can cause the accumulation of mis-spliced mRNAs, including A-RNA, their ability to induce the formation of ZBP1 ligands such as Z-RNA or Z-DNA in cancer cells and/or in the cells of the tumor microenvironment (TME) remains unknown.

Here, we demonstrate that spliceosome inhibition by genetic silencing or small-molecule approaches induces the formation of Z-RNAs in mammalian cells, which act as necroptosis-activating ligands for the host sensor ZBP1.^34–36^ We show that spliceosome inhibitors induce Z-RNA formation and potently activate ZBP1-mediated necroptosis in small cell lung cancer (SCLC) cells and cells of the TME, including cancer-associated fibroblasts (CAFs). Importantly, ZBP1-dependent necroptosis induced by spliceosome inhibition was essential for driving the responsiveness of SCLC tumors to ICB-based immunotherapy. These findings may provide a unique opportunity to activate on-demand necroptosis and trigger innate immune responses in cold cancers, such as SCLC, turning them responsive to ICB-based immunotherapeutic modalities.

## RESULTS

### SF3B1 perturbation triggers the accumulation of endogenous Z-RNAs

**Figure 1.**
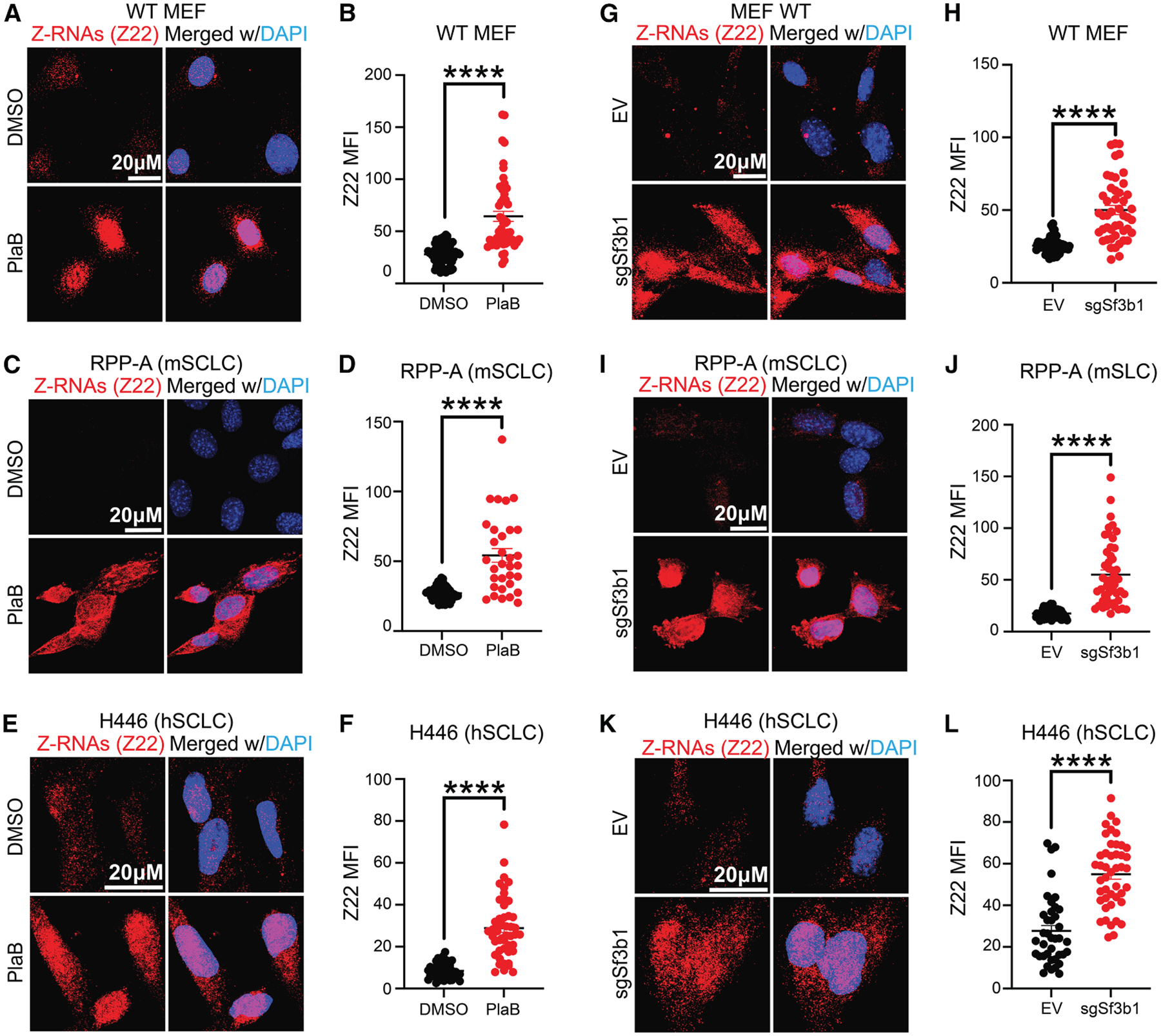
Spliceosome inhibition and SF3B1 depletion induce the accumulation of Z-RNA (A–F) Immunofluorescence (IF) staining images (left) of Z-RNA (red) and DAPI (blue), and quantification of Z-RNA mean fluorescence intensity (MFI) in arbitrary units (right) of (A) and (B) WT MEFs, (C) and (D) mouse (m)SCLC cells (RPP-A), and (E) and (F) human (h)SCLC cells (H446) treated for 18 h with DMSO or PlaB. Scale bars, 20 μM. (G–L) Immunofluorescence staining images (left) of Z-RNA (red) and DAPI (blue), and quantification of Z-RNA MFI (right) of (G) and (H) WT MEF, (I) and (J) RPP-A, and (K) and (L) H446 cells infected with empty vector backbone (EV) or sgSF3b1. Scale bars, 20 μM. CRISPR-Cas9-mediated KD experiments were performed using pooled cell populations after antibiotic selection. All quantification plots of Z-RNA signal intensity are mean ± SEM, two-tailed unpaired Student’s test. *p < 0.05 and ****p < 0.0001. Representative data from at least three independent experiments are shown.

Previous studies have demonstrated that pharmacologic perturbation of the spliceosome, including SF3B1 inhibitors, triggers antiviral signaling and extrinsic apoptosis in cancer cells via dsRNA accumulation, particularly A-RNA.^16,18^ Recent studies elucidated that genetic and pharmacologic perturbation of the spliceosome leads to the production of endogenous Z-NAs in untransformed cells,^37,38^ but whether spliceosome inhibition can lead to Z-NAs in cancer cells and the potential therapeutic implications remain largely unexplored. To evaluate whether spliceosome perturbation may lead to the accumulation of Z-NAs, we first treated immortalized wild-type (WT) MEFs derived from WT mouse with the SF3B1 inhibitor pladienolide B (PlaB)^39–41^ and performed intracellular Z-NA staining by immunofluorescence (IF) and flow cytometry techniques using a Z-RNA/Z-DNA specific antibody (clone Z22).^35,42–44^ MEFs treated with 50 nM PlaB showed significant accumulation of Z-NAs in both the cytoplasm and nucleus, as detected by Z22 antibody through IF and confirmed by intracellular flow cytometry (Figures 1A, 1B and S1A). In addition, immortalized *Zbp1*^−/−^ MEFs (empty vector [EV] MEF) exhibited increased Z22 signal at the same dose of PlaB (Figures S1B and S1D). The signal detected by the Z22 antibody was efficiently reduced following RNase A treatment, suggesting that the Z22 antibody specifically recognizes endogenous Z-RNAs (Figures S1B and S1D). Similarly, RNase H treatment also reduced the intracellular Z22 signal detected upon PlaB treatment (Figures S1E–S1G), suggesting that a subset of Z-NA structures may arise from DNA:RNA hybrids, such as R-loops, consistent with recent findings.^37^ Also, transcription inhibition with actinomycin D abolished the Z22 signal in immortalized *Zbp1*^−/−^ MEFs treated with PlaB, further supporting that endogenous Z-RNAs are the primary sources of Z-NA accumulation after spliceosome perturbation (Figures S2A and S2B).

To extend these studies to cancer cells, we also treated mouse (RPP-A)^45,46^ and human (H446) SCLC cell lines with 50 nM PlaB and observed increased accumulation of Z-RNAs by both IF and flow cytometry (Figures 1C–1F and S1A). To confirm that the accumulation of Z-RNAs was due to on-target inhibition of spliceosome activity, we next tested the effects of genetic depletion of SF3B1 in WT MEFs, H446 (human SCLC), and RPP-A (mouse SCLC) cells. Notably, CRISPR-mediated depletion of SF3B1 significantly increased Z-RNAs levels in all cell lines tested (Figures 1G–1L, and S2C–S2G). Similar to treatment with PlaB or CRISPR-mediated depletion of SF3B1, transfection of siRNAs directed against SF3B1 also induced accumulation of Z-RNAs in WT MEFs (Figures S2H–S2J). Together, these results indicate that spliceosome perturbation through pharmacological inhibition or genetic depletion of SF3B1 triggers the accumulation of left-handed Z-RNAs in mammalian cells.

### Spliceosome perturbation induces the accumulation of Z-RNAs derived from intron-retained RNAs

**Figure 2.**
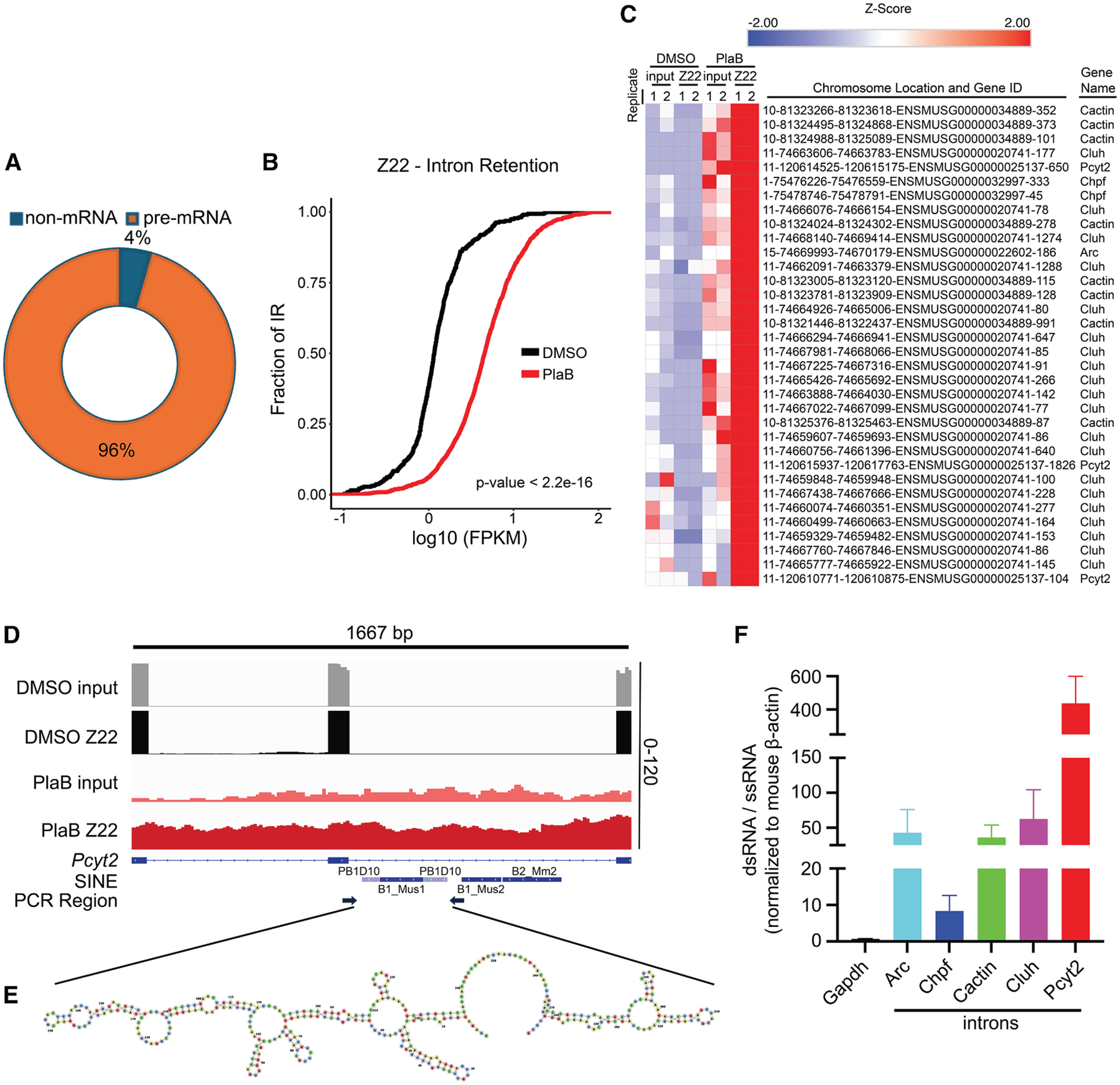
SF3B1 perturbation leads to the accumulation of Z-RNAs originated from intron-retained RNAs (A) Origin of sequenced Z22 RNA immunoprecipitation followed by sequencing (RIP-seq) in EV MEF cells treated with PlaB vs. DMSO. (B) Assessment of intron retention (IR) from RIP-seq analysis in EV MEFs cells following PlaB treatment. IR distribution curve of fraction of IR vs. log_10_FPKM (fragments per kilobase of exon per million mapped fragments) was plotted for DMSO and PlaB treatment groups (*n* = 2 biological replicates). A rightward shift of PlaB treatment group (red) indicates increased IR (*p* < 2.2e-16). Enriched Z-RNA species in PlaB Z22 vs. PlaB input and DMSO Z22 vs. DMSO input were used to calculate the IR graph. (C) Heatmap of *Z* score-normalized FPKM values from RIP-seq analysis showing PlaB-enriched retained introns. Data represent two independent biological replicates. (D and E) Spliceosome inhibition induces intron retention, leading to the formation of double-stranded (ds)RNA secondary structures. (D) Integrative genomics viewer (IGV) image showing the intronic Z22 enrichment in *Pcyt2* gene, and (E) Z22-enriched *Pcyt2* retrotransposon containing introns can form dsRNA secondary structure using dsRNA prediction tool. (F) Introns retained after PlaB treatment can form dsRNA structures. RNA lysates from EV MEFs ± PlaB were treated ± RNaseONE, a single-stranded (ss) RNA-specific ribonuclease, followed by RT-qPCR (mean ± SEM, *n* is equal to or greater than three biological replicates). Relative dsRNA/ssRNA fold changes of PlaB-treated over DMSO-treated samples were normalized to *ActB* mRNA. *Gapdh*, a gene not expected to form dsRNA under normal conditions, was used as the control for non-dsRNA-forming transcripts in the two experimental conditions tested, PlaB and DMSO.

To investigate the genomic sources of Z-RNAs accumulated in response to spliceosome perturbation, we used the Z22 antibody to immunoprecipitate Z-RNA from PlaB- and DMSO-treated *Zbp1*^−/−^ MEFs (EV MEFs) and performed RNA immunoprecipitation sequencing (RIP-seq). Sequencing Z22-enriched RNAs showed that most (>95%) RNA species enriched after PlaB treatment were precursor mRNAs (pre-mRNA); other non-mRNA species constituted the remainder of the reads, further supporting that Z-RNAs are the primary source of Z-NA accumulation after spliceosome perturbation (Figures 2A and S3A; Table S1). Given that spliceosome inhibition can lead to widespread intron retention,^18,47,48^ we sought to determine whether the Z-RNAs that accumulate following spliceosome inhibition originate from intron-retained RNAs. RIP-seq analysis revealed a significant increase in intron retention in PlaB-treated cells compared to DMSO control (Figure 2B; Table S2). Notably, genes with intron retention were significantly enriched in Z22 pull-downs from PlaB-treated samples when compared to DMSO control (Figures 2C and S3B; Tables S3 and S4). Among these intron-retained genes were *Pcyt2*, *Cactin*, and *Cluh*, which contain SINE (short interspersed nuclear elements) elements potentially capable of contributing to the formation of double-stranded (ds) secondary structures (Figures 2D, 2E, and S3C–S3F). Similarly, we found that introns without retrotransposons were also enriched in Z22 pull-downs from PlaB-treated cells and were predicted to form ds secondary structures (Figures S3G–S3J), suggesting that transcriptome-wide intron retention may contribute to the formation of Z-RNAs after spliceosome inhibition. To further validate that these Z22-enriched retained introns identified in PlaB-treated cells can form dsRNA structures, we next isolated RNA from PlaB- and DMSO-treated cells and performed single-stranded RNA (ssRNA) digestion followed by RT-qPCR. We found that Z22-enriched introns in PlaB-treated cells, compared to DMSO control cells, were significantly more resistant to ssRNA digestion than a well-characterized ssRNA gene (*Gapdh*) (Figure 2F), supporting the capability of these introns retained after spliceosome inhibition to form dsRNA structures. Collectively, these data indicate that spliceosome perturbation induces global RNA intron retention, which contributes to the formation of Z-RNAs.

### Spliceosome inhibition activates ZBP1-driven cell death

**Figure 3.**
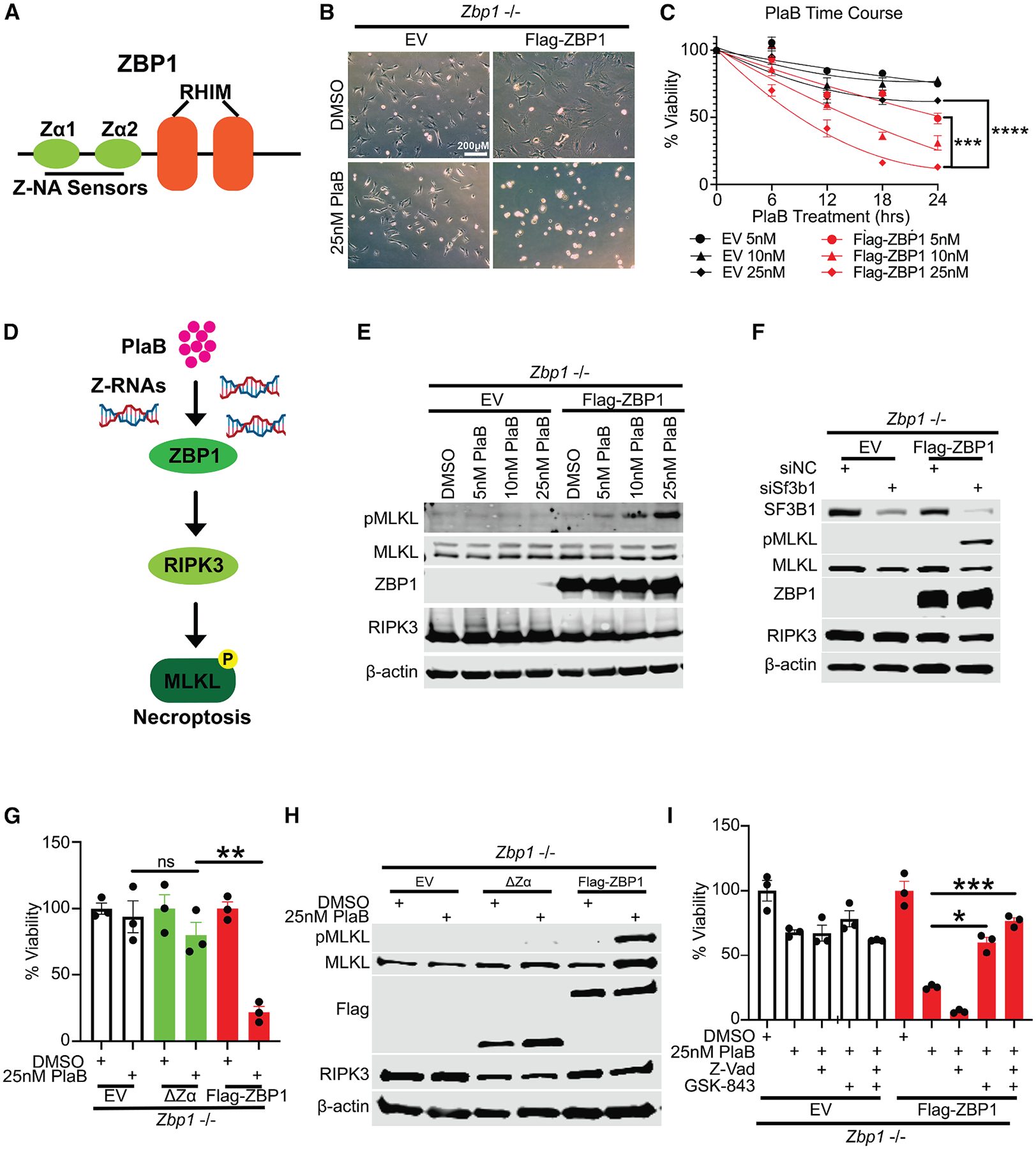
Spliceosome inhibition induces ZBP1-dependent cell death (A) Schematic of ZBP1 protein structure. (B) Brightfield microscope images of *Zbp1*^−/−^ MEFs expressing EV or Flag-ZBP1 after 18 h of PlaB treatment. Scale bars, 200 μM. (C) Viability kinetics of EV and Flag-ZBP1 MEF cells following PlaB treatment over 24 h. (D) Schematic of the proposed model for PlaB-mediated necroptosis (pMLKL expression). (E) Immunoblots of dose-dependent PlaB-induced necroptosis in EV and FLAG-ZBP1 MEFs after 18 h PlaB treatment. (F) Immunoblots of MLKL activation (pMLKL) after 72 h of siRNA *Sf3b1* KD in EV and FLAG-ZBP1 MEFs. (G) Cell viability after 18 h of PlaB treatment in EV, ΔZα mutant, and FLAG-ZBP1 MEFs. (H) Immunoblots showing the activation of MLKL (pMLKL) after 18 h of PlaB treatment in EV, ΔZα mutant, and FLAG-ZBP1 MEFs. (I) Cell viability in EV and FLAG-ZBP1 MEFs after 18 h of PlaB, Z-Vad (50 μM), or RIPK3 inhibitor (GSK-843, 5 μM) treatments. All viability graphs are mean ± SEM, two-tailed unpaired Student’s test. *p < 0.05, **p < 0.01, ***p < 0.001, and ****p < 0.0001. Representative data from at least three independent experiments are shown.

Recent studies reported that viral Z-NAs derived from influenza A virus or curaxin treatment act as ligands for the host sensor ZBP1. ZBP1 can recognize both Z-RNA and Z-DNA duplexes through its Zα domains, initiating ZBP1-dependent necroptosis and culminating in a highly immunogenic cell death (Figure 3A).^34–36^ To evaluate whether spliceosome inhibition is capable of triggering ZBP1-dependent cell death in mammalian cells, we exposed immortalized *Zbp1*^−/−^ MEFs stably expressing Flag-ZBP1 or an EV to PlaB treatment. Remarkably, PlaB induced potent ZBP1-dependent cell death in Flag-ZBP1 MEFs within 24 h at doses correlating with its capacity to trigger Z-RNA formation (Figures 3B, 3C, and S4A–S4D), suggesting that ZBP1 expression may contribute to cell death caused by spliceosome inhibition. Upon activation, ZBP1 forms a complex with RIPK3, and subsequently, RIPK3 phosphorylates MLKL (mixed lineage kinase domain-like), leading to necroptosis (Figure 3D).^49,50^ Indeed, PlaB-induced cell death triggered ZBP1-dependent phosphorylation of MLKL in FLAG-ZBP1 MEFs, but not in MEFs lacking ZBP1 (EV MEFs), demonstrating that it is ZBP1-dependent necroptosis (Figure 3E). To validate these results in a more physiological context, we also treated WT MEFs derived from WT mouse with PlaB and confirmed MLKL phosphorylation (Figure S4E). Moreover, siRNA-mediated knockdown (KD) of SF3B1 mimicked the effects of pharmacological inhibition of the spliceosome and induced MLKL phosphorylation in FLAG-ZBP1 MEFs and WT MEFs, but not in EV MEFs (Figures 3F and S4F–S4J). Next, we asked whether the Zα domains of ZBP1 involved in Z-NA sensing were required to trigger ZBP1-driven necroptosis. Of note, 18 h of PlaB treatment in cells reconstituted with ZBP1 mutant lacking Zα domains (ΔZα) did not result in such cell death (Figures 3 and S4K). Accordingly, while PlaB-induced cell death was accompanied by ZBP1-dependent phosphorylation of MLKL in FLAG-ZBP1 MEFs, phospho-(p)MLKL was completely abrogated in ΔZα MEF mutants (Figure 3H), confirming that Z-RNA sensing is required to trigger ZBP1-dependent necroptosis after spliceosome inhibition.

To further understand the cell death mechanism induced by SF3B1 inhibition, we next co-treated EV and Flag-ZBP1 MEFs with a pan-caspase inhibitor (Z-VAD), a RIPK3 inhibitor (GSK-843), or a combination of both compounds in the presence of PlaB. We observed that PlaB-mediated cell death was rescued by co-treatment with GSK-843 alone or the combination of Z-VAD and GSK-843 (Figures 3I and S4L), suggesting that RIPK3 is required for cell death induced by SF3B1 inhibition and that necroptosis is the primary mode of cell death in our system. Interestingly, Z-VAD alone increased PlaB-mediated cell death (Figure 3I), which is consistent with previous findings that allowed ZBP1-RIPK3-MLKL-mediated necroptosis to proceed unchecked after pan-caspase inhibition.^36^ Altogether, these findings indicate that ZBP1 senses endogenous Z-RNAs accumulated upon spliceosome perturbation to activate RIPK3-dependent cell death, illuminating a novel mechanism by which STTs can trigger innate immune responses in mammalian cells competent for ZBP1-driven and RIPK3-driven death signaling.

### Spliceosome perturbation activates ZBP1-mediated cell death in necroptosis-competent SCLC cells

**Figure 4.**
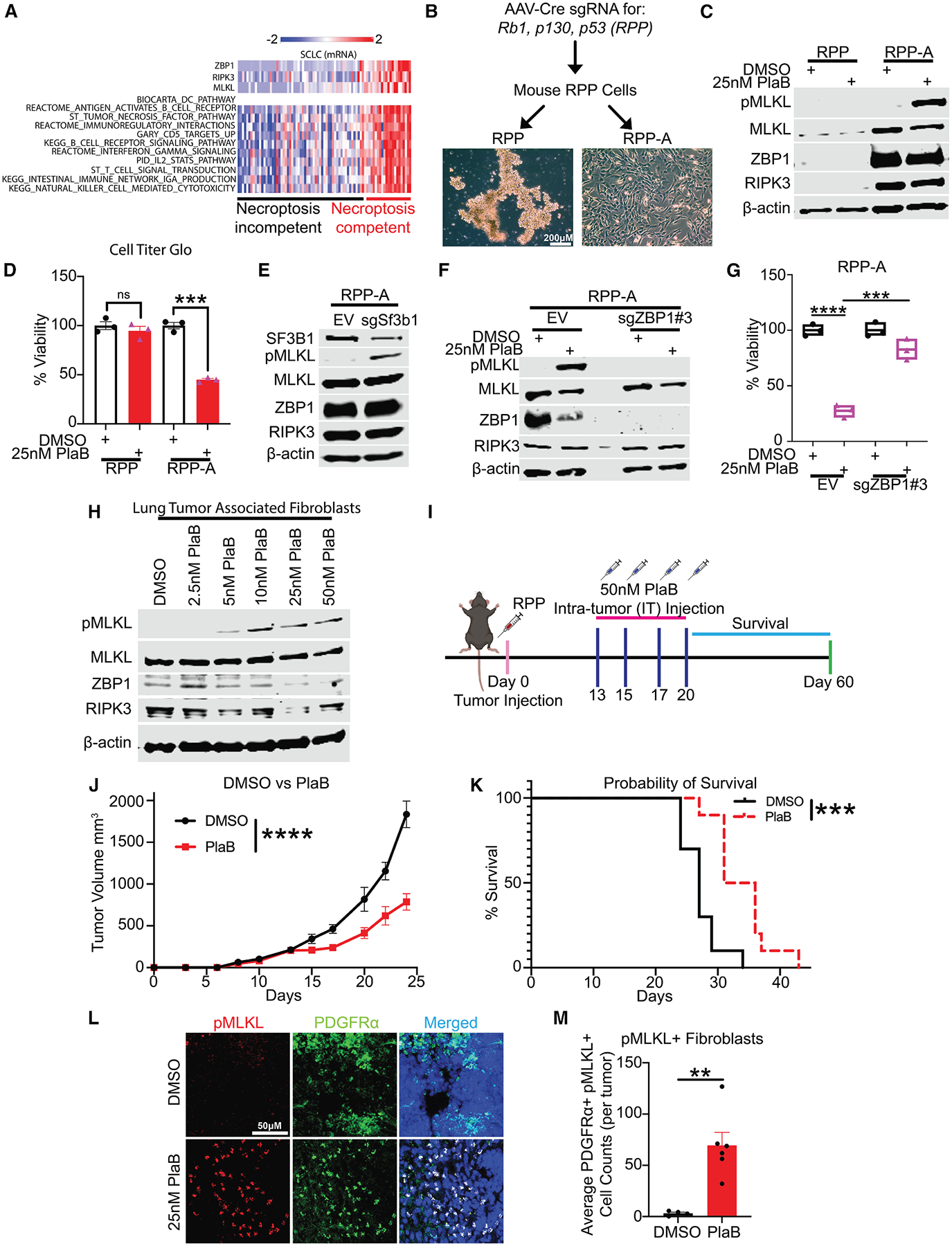
Spliceosome perturbation triggers ZBP1-driven cell death in necroptosis competent SCLC cells and CAFs, leading to decreased SCLC tumor growth *in vivo* (A) Heatmap of *ZBP1*, *MLKL*, and *RIPK3* gene expression from human SCLC dataset (George et al., Nature, 2015)^65^. (B) Top: schematic of the generation of the mSCLC model (RPP). Bottom: phase contrast images of isogenic mouse SCLC cell lines. Scale bars, 200 μM. (C) Immunoblots of pMLKL and key necroptosis proteins (endogenous MLKL, RIPK3, and ZBP1) in RPP and RPP-A after 18 h of PlaB treatment. (D) Cell viability of RPP and RPP-A after 18 h of PlaB treatment. (E) immunoblots showing the KD of Sf3b1 and the activation of MLKL (pMLKL) in RPP-A cells using CRISPR-Cas9 methodology. CRISPR-Cas9-mediated KD experiments were performed using pooled cell populations after antibiotic selection. (F) Immunoblots of MLKL activation (pMLKL) in parental RPP-A cells and RPP-A cells lacking ZBP1. Experiments were performed using a single-cell clone derived from sg*Zbp1* #3-mediated knockout of Zbp1. (G) Cell viability analysis in parental RPP-A cells and RPP-A cells lacking ZBP1 after 18 h of PlaB treatment. Experiments were performed using single-cell clone derived from sg*Zbp1* #3-mediated knockout of Zbp1. (H) Immunoblots of dose-dependent activation of MLKL (pMLKL) in lung tumor-associated fibroblasts after 18 h of PlaB treatment. (I) Schematic of *in vivo* experimental design to measure tumor growth and survival in RPP tumors following DMSO or PlaB treatment. (J and K) Intratumoral administration of PlaB reduces RPP tumor growth and prolongs survival. (J) Tumor growth curves of RPP SCLC cells inoculated into syngeneic C57BL/6J mice treated with DMSO or PlaB (*n* = 10) and (K) Kaplan-Meier survival curve from mice in (J). (L and M) Spliceosome inhibition triggers necroptosis in CAFs. (L) Immunofluorescent staining of pMLKL and PDGFRα in representative tumors treated with DMSO or PlaB, and (М) quantification of pMLKL and PDGFRα from tumors indicated in L (*n* = 6). Scale bars, 20 μM. All quantitative graphs are mean ± SEM, two-tailed unpaired Student’s test. Not significant (ns), **p < 0.01, ***p < 0.001, and ****p < 0.0001. Representative data from at least three independent experiments are shown. *p* values were obtained using a two-way ANOVA test (J) and a log rank test (K), ***p < 0.001 and ****p < 0.0001.

To expand our findings to cancer, we focused on SCLC, a classical immunologically cold tumor that, despite advancements in cancer immunotherapy, remains a recalcitrant disease.^51–59^ Since loss of necroptosis machinery is a common occurrence in cancer cells,^60–64^ we explored publicly available datasets with transcriptomic data of SCLC human tumors^65^ and cell lines and found that about 30% of SCLC cases and cell lines express the necroptosis machinery (ZBP1, RIPK3, and MLKL) (“necroptosis competent”). Of note, these tumors were associated with interferon (IFN) signaling and immune activation signatures (Figure 4A). However, a majority of SCLC tumors and cell lines expressed necroptosis components at low levels or did not express one or more components of the necroptosis machinery (“necroptosis incompetent”), and were inversely associated with immune-related signatures, suggesting a cold tumor microenvironment (Figures 4A and S5A). To test the capacity of SF3B1 inhibitors to induce ZBP1-driven necroptosis in SCLC cells, we used the mouse SCLC cell lines established from a CRISPR-derived *Tp53*, *Rb1*, *Rbl2* (RPP) SCLC genetically engineered mouse model.^24,45,46,66^ We used RPP cells, classical neuroendocrine SCLC cells, and an isogenic non-neuroendocrine adherent derivative, RPP-A cells^66^ (Figure 4B). Interestingly, while RPP cells lacked expression of the components of necroptosis machinery, RPP-A cells exhibited high endogenous levels of MLKL, RIPK3, and ZBP1 expression, suggesting a necroptosis competent SCLC cell line (Figure 4C). Accordingly, PlaB treatment or depletion of SF3B1 induced phosphorylation of MLKL in necroptosis competent RPP-A cells, as well as a significant decrease in cell viability, whereas RPP-treated cells remained viable throughout the duration of the experiment (Figures 4C–4E, S5B, and S5C). Similarly, treatment with the classical necroptosis stimulus of tumor necrosis factor (TNF, 20 ng/mL), combined with cycloheximide (CHX, 250 ng/mL) and the caspase inhibitor Z-VAD-fmk (CZ, 50 μM)^34,67^ induced MLKL phosphorylation and a dramatic decrease in cell viability in RPP-A cells (Figures S5D and S5E). Given that PlaB can inhibit additional components of the SF3b complex, including SF3B3,^40^ we next sought to investigate whether depletion of other SF3b subunits similarly impacts necroptosis. Strikingly, CRISPR-Cas9-mediated deletion of any core subunits of the SF3b complex induced p-MLKL (Figures S5F–S5M), further supporting the idea that spliceosome inhibition triggers necroptosis. Importantly, genetic ablation of ZBP1 prevented MLKL activation and the decrease in cell viability caused by spliceosome inhibition in RPP-A cells, indicating that ZBP1 is essential for PlaB-induced necroptosis (Figures 4F, 4G, and S5N). Together, these findings suggest that spliceosome inhibition can induce ZBP1-driven necroptosis in SCLC cells.

### Spliceosome inhibition activates ZBP1-dependent cell death in tumor-associated fibroblasts, induces immune cell infiltration, and decreases tumor growth

Since most SCLC tumors and cell lines exhibited low or absent expression of key components of the necroptosis machinery (Figures 4A and S5A), we investigated whether spliceosome inhibitors could target other necroptosis-competent cells within the TME. To our knowledge, the potential effects of spliceosome inhibition on the TME remain completely unexplored. Importantly, a previous study demonstrated that CAFs are necroptosis competent and that therapeutic induction of ZBP1-driven necroptosis in CAFs enhanced ICB response in mouse melanoma models.^36^ Thus, we sought to determine whether SF3B1 inhibition triggers ZBP1-driven necroptosis in human lung cancer CAFs. Consistent with previous results, 18-h treatment with PlaB efficiently induced MLKL phosphorylation in CAFs derived from patients with lung cancer and pancreatic cancer, suggesting that these cells are necroptosis-competent and that spliceosome perturbation can trigger necroptosis in patient-derived CAFs (Figures 4H and S6A). Furthermore, these results were accompanied by an accumulation of Z-RNAs in CAFs treated with PlaB, as determined by IF staining with Z22 antibody (Figures S6B–S6E). To examine whether the necroptosis induced by PlaB treatment in lung fibroblasts is driven by ZBP1, we next isolated primary lung fibroblasts from WT and *Zbp1*^−/−^ mice and exposed them to PlaB. Notably, PlaB treatment induced MLKL phosphorylation and cell death in primary lung fibroblasts derived from WT mice, while this phenotype was rescued in fibroblasts from *Zbp1*^−/−^ mice (Figures S6F and S6G), suggesting that PlaB induces ZBP1-driven necroptosis in lung fibroblasts. These findings suggest the hypothesis that the induction of necroptosis through spliceosome perturbation in CAFs from the SCLC TME would trigger antitumor responses even if the cancer cells are not necroptosis-competent. To test this, we generated SCLC syngeneic tumors in C57BL/6J mice using the necroptosis-incompetent SCLC cell line RPP, and when tumors became palpable, we intratumorally injected four doses of 50 nM PlaB every 2–3 days (Figure 4I). PlaB significantly reduced tumor burden and increased survival (Figures 4J, 4K, and S7A), which is in agreement with a previous study on ovarian cancer.^68^ Strikingly, we found a significant increase in MLKL phosphorylation in tumor sections from PlaB-treated tumors, including in CAFs, as confirmed by co-staining with the fibroblast marker PDGFRα (platelet-derived growth factor receptor alpha),^36^ (Figures 4L and 4M), suggesting that spliceosome inhibition can induce necroptosis *in vivo* in SCLC tumor fibroblasts. Next, to better understand the immune consequences of PlaB treatment *in vivo*, we evaluated tumor immune infiltration by flow cytometry analysis. While PlaB treatment did not induce significant changes in immune cells expressing CD4, CD8, Cd11b, or F4/80 markers, we observed a striking increase in immune cells expressing NK1.1 and γδT-cells (Figures S7B–S7J). Indeed, IF analysis of tumor sections confirmed enhanced infiltration of natural killer (NK) cells as well as γδT-cells in PlaB-treated tumors (Figures S7K–S7N), suggesting that spliceosome inhibition enhances immunogenicity *in vivo*. Together, our data demonstrate that spliceosome inhibition effectively induces ZBP1-driven necroptosis in CAFs from SCLC tumors and modulates the immunosuppressive SCLC TME, warranting further investigation into the interaction between necroptosis and immune cell infiltration.

### Spliceosome inhibition enhances response to ICB therapy in SCLC models and requires ZBP1 expression

**Figure 5.**
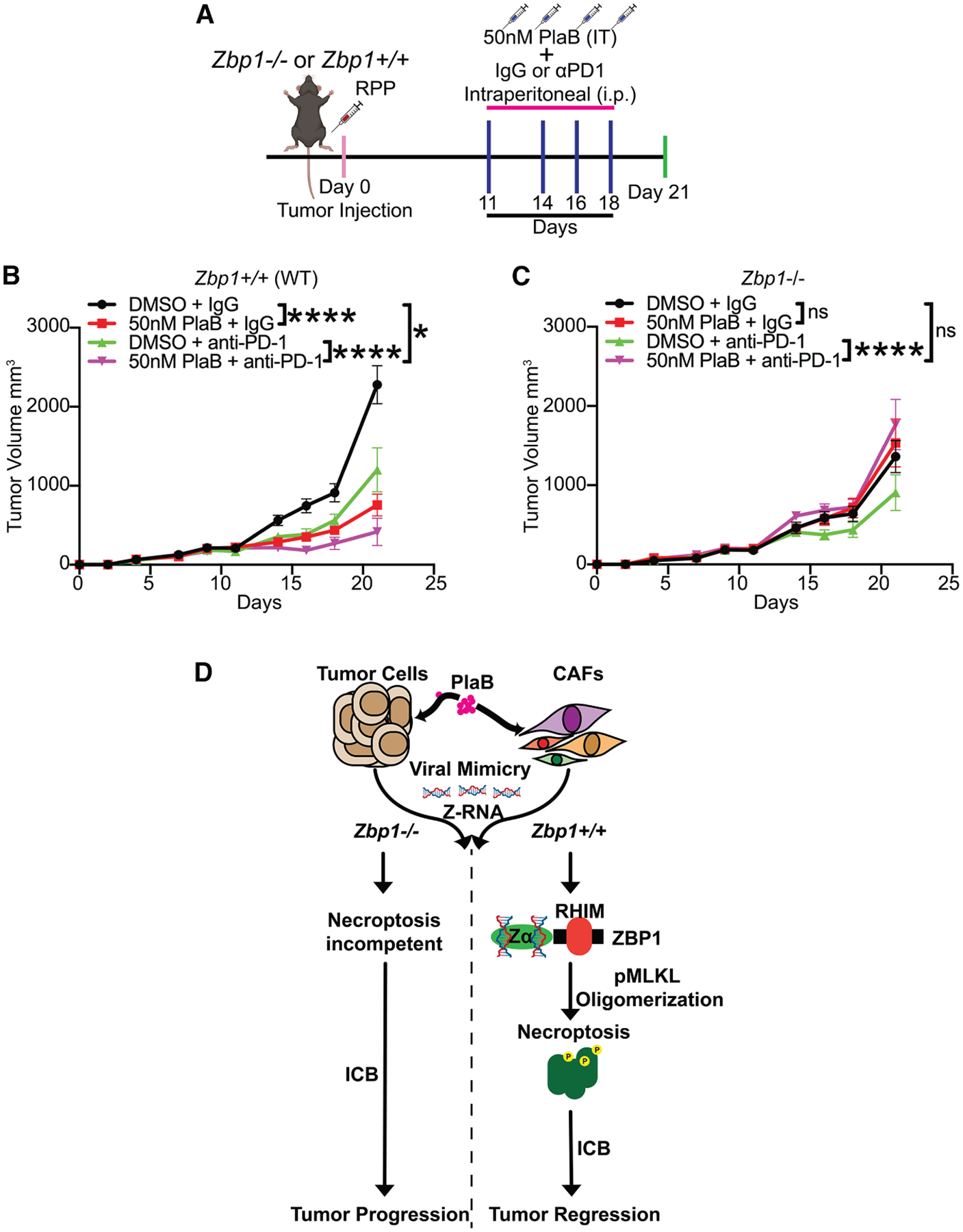
Inhibition of SF3B1 improves the efficacy of ICB therapy in SCLC models and is dependent on ZBP1 expression (A) Schematic of the *in vivo* experimental design to assess tumor growth and survival in RPP tumors following PlaB, anti-PD1 antibody, or their combination in C57BL/6J mice with either *Zbp1*^+/+^ (B) or *Zbp1*^−/−^ (C) genetic backgrounds. (B and C) ZBP1 expression is required for PlaB to effectively reduce tumor volume and enhance the response to immunotherapy. (B) Tumor growth curves of RPP SCLC cells inoculated into syngeneic C57BL/6J *Zbp1*^+/+^ mice treated with PlaB, anti-PD1 antibody, or their combination (*n* = 14) in accordance with the indicated schedule (A). (C) Tumor growth curves of RPP SCLC cells inoculated into syngeneic C57BL/6J *Zbp1*^−/−^ mice treated with PlaB, anti-PD1 antibody, or their combination (*n* = 14) in accordance with the indicated schedule (A). (D) Z-RNA accumulation and ZBP1-dependent necroptosis induced by spliceosome inhibition decrease tumor growth and enhance immunotherapy response in mouse models of SCLC. Importantly, the therapeutic effects of PlaB treatment *in vivo* are entirely dependent on ZBP1 expression. Thus, spliceosome inhibition represents a therapeutic strategy to trigger on-demand ZBP1-dependent necroptosis to enhance immunotherapy response in cancers that are hard to treat. *p* values were obtained using a two-way ANOVA test for data indicated in (B) and (C), not significant (ns), *p < 0.05 and ****p < 0.0001.

A previous study suggests that spliceosome inhibition can enhance immunotherapy in certain cancer models.^68^ Given that PlaB treatment induced ZBP1-driven necroptosis in CAFs and increased intratumoral infiltration of NK cells and γδT-cells in SCLC tumors (Figures S7K–S7N), we next sought to leverage these findings for cancer immunotherapy. To test whether ZBP1-driven necroptosis triggered by PlaB in CAFs was able to enhance ICB response, we generated RPP SCLC syngeneic tumors in either *Zbp1*^+/+^ (WT) or *Zbp1*^−/−^ mice. We treated these mice with four cycles of intratumoral injections of PlaB or DMSO in combination with intraperitoneal administration of anti-PD-1 antibody or isotype IgG control antibody (Figure 5A). Strikingly, PlaB treatment significantly enhanced the sensitivity of RPP tumors to anti-PD1 blockade therapy only in WT mice but not in *Zbp1*^−/−^ mice (Figures 5B and 5C). Furthermore, while PlaB treatment alone significantly decreased tumor growth in WT mice, this effect was completely rescued in *Zbp1*^−/−^ mice (Figure 5C). These findings demonstrate that ZBP1 expression is required for spliceosome inhibition to induce anti-tumor effects and enhance the efficacy of ICB-based immunotherapy in SCLC syngeneic models (Figure 5D). This underscores spliceosome perturbation as a promising strategy to therapeutically induce necroptosis to turn cold tumors hot.

## DISCUSSION

Recent studies reported that small-molecule inhibitors of the spliceosome showed potent antitumor activity through the accumulation of endogenous dsRNA, leading to an antiviral immune response.^17,18^ However, mechanistically, how spliceosome inhibition leads to antitumor immunity remains poorly understood. In addition, while most studies on tumor-intrinsic immune responses via dsRNA-sensing pathways focus on the A-form dsRNA (A-RNA),^18^ endogenous dsRNAs can also adopt the less common left-handed conformation (Z-RNA).^36^ These Z-RNAs remain largely unexplored in the context of cancer but hold significant potential to induce necroptosis, a highly immunogenic form of cell death.^35,69,70^ Here, we demonstrate that spliceosome perturbation, via genetic depletion or inhibition of SF3B1, results in the aberrant accumulation of endogenous Z-RNAs and activation of the Z-RNA sensor ZBP1, ultimately triggering necroptosis in cells within the TME, providing a novel mechanism linking spliceosome inhibition to necroptosis to cancer immunotherapy. Our findings demonstrate that spliceosome inhibition induces widespread RNA intron retention across diverse genomic regions, promoting the formation of Z-conformation dsRNA secondary structures. Notably, inducing ZBP1-driven necroptosis through spliceosome inhibition within the TME not only activates antitumor immune responses but also significantly potentiates ICB efficacy in mouse syngeneic models of SCLC. Indeed, ZBP1 was responsible for the bulk of rekindled responsiveness to ICB, demonstrating that STT-induced Z-RNA, not A-RNA, drives therapy responsiveness.

One of the key challenges in addressing poor immunotherapy responses in certain cancers resides in their immunologically “cold” nature.^66,71–75^ We and others have shown that triggering innate immune responses within the TME can effectively induce antitumor immune responses in cold tumors, such as SCLC.^22–24,36^ Our data indicate that spliceosome inhibition can trigger ZBP1-driven necroptosis in necroptosis-competent SCLC cells, offering a promising avenue for therapeutic intervention. However, cancer cells often disable innate-immune and cell-death signaling pathways, including key components of the necroptosis machinery, since it provides a survival advantage during tumorigenesis.^60,61^ This adaptive mechanism may limit the ability of spliceosome inhibitors to effectively trigger immunogenic cell death in cancer cells. Notably, our study revealed that spliceosome inhibitors can trigger ZBP1-dependent necroptosis in CAFs from the TME, which retain intact necroptosis pathways, not only reducing tumor growth but also enhancing ICB responses in SCLC syngeneic models. These discoveries have important implications for inducing viral mimicry in “cold” tumors, since spliceosome inhibitors may mediate antitumor immunity by acting on any necroptosis-competent cells, including cells of the TME. Thus, spliceosome inhibitors might induce a more immunogenic TME in all patients with SCLC, regardless of whether SCLC cells have compromised necroptosis signaling. Importantly, these results align with findings from our previous work,^36^ who identified an anti-cancer small molecule, curaxin, which activates ZBP1 by inducing Z-DNA formation in fibroblasts, sensitizing melanoma cells to immunotherapy. Importantly, the fact that ZBP1 expression is induced by IFN^76–79^ and that many tumors exhibit IFN-associated signatures,^80,81^ suggests that spliceosome inhibitors may preferentially induce necroptosis in cancer cells and in non-malignant cells within the TME (e.g., CAFs), where IFN signaling is often active and ZBP1 expression is most likely upregulated.

In summary, our study uncovers a previously unrecognized mechanism by which spliceosome inhibition induces ICB responsiveness in cold tumors, entirely dependent on ZBP1 expression. Our findings demonstrate that intron-retained RNAs generated by spliceosome inhibition act as potent necroptosis-activating ligands for the host immune sensor ZBP1, providing a unique opportunity to therapeutically restore immune responses in immunologically “cold” tumors.

### Limitations of the study

Our study demonstrates that spliceosome inhibition induces the accumulation of Z-NAs, triggering ZBP1-mediated cell death in both tumor cells and CAFs, ultimately enhancing anti-tumor immunity and immunotherapy responsiveness. Necroptosis-competent cells exhibited reduced viability and pMLKL activation upon PlaB treatment, whereas necroptosis-incompetent cells lacking Zbp1 and Ripk3 were unaffected. Consistently, cells lacking ZBP1 or expressing Z-NA-sensing mutants failed to undergo necroptosis or pMLKL activation after PlaB treatment, supporting a mechanistic link between PlaB’s antitumor effects and ZBP1-mediated necroptotic signaling. However, we acknowledge that ZBP1 and RIPK3 play crucial roles in multiple biological processes, and it remains inconclusive to attribute cell death exclusively to necroptosis. Future studies using Ripk3^−/−^ and Mlkl^−/−^ mice will be required to rigorously delineate the cell death mechanisms induced by spliceosome inhibition in this context. In addition, the effects of spliceosome inhibition on other cell types within the TME remain uncharacterized. Whether ZBP1-driven cell death in these additional stromal or immune cell populations contributes to enhanced immunotherapy responses is still unknown and warrants further investigation. Lastly, although we observe ZBP1-dependent cell death in “necroptosis-competent” SCLC cells, it is well-established that many cancers suppress necroptotic signaling to evade cell death. Therefore, further research is needed to understand how ZBP1-dependent cell death can be reactivated therapeutically in necroptosis-deficient tumors to expand the applicability of this approach.

## RESOURCE AVAILABILITY

### Lead contact

Requests for information and resources should be directed to the lead contact Israel Cañadas, israel.canadas@fccc.edu.

### Materials availability

All uniquely generated cell lines in this study are available from the lead contact.

### Data and code availability

- All high-throughput RIP-seq data were deposited in the public functional genomics data repository GEO (https://www.ncbi.nlm.nih.gov/geo/), and the accession number is listed in the key resources table.
- No custom code was generated.

## STAR★METHODS

### EXPERIMENTAL MODEL AND STUDY PARTICIPANT DETAILS

#### Mice

Six-to-eight-weeks-old male and female mice were used for all tumor studies. *Zbp1*^−/−^ breeding mice were obtained from Dr. Siddharth Balachandran and used for *in vivo* tumor studies that have been previously described.^90^ For the experiments do not require *Zbp1*^−/−^ mice, C57BL/6J mice were obtained from the Jackson Laboratory (JAX; 000664) and were allowed to acclimate to the housing conditions at the Fox Chase Cancer Center (FCCC) for at least 1 week before experiments. All mice experiments were performed under protocols approved by Institutional Animal Care and Use Committee at FCCC and housed under specific pathogen-free environments. The animal experiments were not blinded, and no statistical methods were used to predetermine the group sizes. The mice were randomized into control and experimental groups.

#### Cell lines and cell culture

The human SCLC cell line NCI-H446 was obtained from the American Type Culture Collection (ATCC). Mouse isogenic RPP and RPP-A SCLC genetically engineered mouse models (GEMM) were obtained from Dr. Matthew Oser. Immortalized Wild-type (WT), EV, ΔZα, and Flag-ZBP1 mouse embryonic fibroblasts (MEFs) were obtained from Dr. Balachandran Siddharth. Primary *Zbp1*^+/+^ and *Zbp1*^−/−^ MEFs were obtained from Dr. Siddharth Balachandran. Primary lung *Zbp1*^+/+^ and *Zbp1*^−/−^ fibroblasts were isolated from six-to-eight-weeks-old *Zbp1*^+/+^ and *Zbp1*^−/−^ mice. Human patient cancer-associated fibroblasts (CAFs): Pancreatic CAFs and Lung CAFs cell lines were obtained from Dr. Edna Cukierman. All human SCLC cell lines were cultured in RPMI-1640 containing 10% fetal bovine serum (FBS, Hyclone), 1.5 g/L Sodium bicarbonate, 4.5 g/L glucose, 2 mM L-Glutamine, 10 mM HEPES, 1 mM Sodium pyruvate, and 1X penicillin-streptomycin. Immortalized MEFs, Pancreatic CAFs, Lung CAFs, primary MEFs, and primary lung fibroblasts cell lines were cultured in DMEM containing 10% FBS, 1.5 g/L Sodium bicarbonate, 4.5 g/L High Glucose, 2 mM L-Glutamine, 1 mM Sodium pyruvate, and 1X penicillin-streptomycin. Isogenic RPP and RPP-A murine SCLC cell lines were cultured in human SCLC cell line medium supplemented with HITES (1X insulin-transferrin-selenium, 10 nM β-estradiol, and 10 nM hydrocortisone). All cells were cultured in a humidified incubator at 37°C with 5% CO_2_ and routinely tested for mycoplasma.

### METHOD DETAILS

#### Proliferation assay

Equal numbers of cells were plated in 12-well plates and cultured with respective medium at 37°C with 5% CO_2_. Cells were treated with DMSO or Pladienolide B (PlaB) (Cayman Chemicals; 16538) at varying doses for 6, 12, 18, or 24 h and viable cells were counted. Percent viability was normalized to DMSO treatment group.

#### Immunofluorescence (IF) microscopy

Cells were plated on 8-well Lab-Tek II Chamber Slide with Cover (Thermo Fisher; 154941) and allowed to adhere before experimentation. Following treatment, cells were washed with PBS and fixed for 10 min with 4% paraformaldehyde (PFA) in PBS (Themro Fisher; J61899). Followed by three washes in PBS and permeabilized with 0.5% (v/v) Tritox X-100 in PBS for 15 min. Then blocked with MAXblock blocking medium (Active Motif; 102224) for 1 h at 37°C and followed by primary antibodies incubation with Z-Nucleic acid antibodies (Z22; Absolute Antibody; Ab00783–23.0) at 4°C for overnight. Followed by three washes in PBS and incubated with fluorophore-conjugated secondary antibodies (Thermo Fisher; A32790) for 1 h at room temperature. Followed by additional three washes in PBS, and then the slides were mounted in Prolong Gold antifade mountant with DNA stain DAPI (Thermo Fisher; P36935). Images were taken with Leica SP8 confocal microscope and analyzed with ImageJ software. For immunofluorescent staining of tumor tissues, live tumors were preserved with fresh 4% PFA (Themro Fisher; J61899) overnight at 4°C. Followed by 30% sucrose cryopreservation, then sectioned to 12μМ thickness using a histology cryostat. Tumor sections are washed three time in PBS, and permeabilized with 0.5% (v/v) Triton X-100 (Sigma; X100–500ML) in PBS for 15 min at room temperature. Tumors were incubated at 37°C for 1 h in MAXblock blocking medium (Active Motif; 102224), and then probed with primary antibodies overnight at 4°C. After three washes in PBS, tumor sections were stained with fluorophore-conjugate secondary antibodies for 1 h at room temperature. Followed by three washes in PBS, tumor sections were mounted in ProLong Gold antifade mountant with DAPI (Thermo Fisher; P36935). Immunofluorescence images were taken with Leica SP8 confocal microscope and analyzed with ImageJ software. When required, slides were treated with RNase A at the concentration of 1 mg/mL (Thermo Fisher; EN0531) and RNase H at the concentration of 20U/mL (Thermo Fisher; 18021014) prior to primary antibody incubation for 1 h at 37°C. The following primary antibodies were used for tumor section immunofluorescence studies: pMLKL (Abcam; ab196436), NK1.1 (Thermo Fisher; MA1–70100), TCR γ/δ (BioLegend; 118118), and PDGFRα (Invitrogen; 14-1401-82).

#### Intracellular Z-RNA flow cytometry staining

Treated cells were collected and washed with PBS followed by 10 min incubation with Zombie Green Fixable Viability dye (BioLegend; 423112). Cells were washed with PBS and followed by 10 min fixation with 4% PFA in PBS (Thermo Fisher; J61899). Followed by three washes in PBS and permeabilized with 0.5% (v/v) Tritox-X-100 in PBS for 15 min. Subsequently, cells were washed with PBS followed by primary antibodies incubation with Z-Nucleic Acid antibodies (Z22; Absolute Antibody; Ab00783–23.0) for 1 h. Followed by three washes in PBS and incubated with fluorophore-conjugate secondary antibodies (Thermo Fisher; A32754) for 1 h. Followed by three addition washes in PBS.

#### Immunoblotting

Cells were lysed in Pierce RIPA Buffer (Thermo Fisher; 89900) with protease (Thermo Scientific; 78430) and phosphatase inhibitors (Thermo Scientific; 78428). Cell lysates were incubated on ice for 30 min and then briefly sonicated. Protein concentrations were quantified using Pierce BCA Protein Assay Kit (Thermo Fisher; 23225) according to the manufacturer’s instructions. Samples containing equal volume and protein extracts were then heated to 95°C for 5 min in 6X Laemmli sample buffer containing 10% β-mercaptoethanol. 30 μg–40 μg of total protein extracts were run on freshly made SDS-Page gel (10%) and the gels were then transferred onto a 0.45 μm nitrocellulose membrane (Bio-Rad; 1620115) using Bio-Rad Wet Tank Blotting System (Bio-Rad; 1658034).

The membranes were briefly washed in dH2O, then Tris-buffered saline (diluted 1X-TBS) (Bio-Rad; 1706435), and blocked in 5% BSA in 1X-TBS supplemented with Tween 20 (0.1% v/v, TBST) (Thermo Scientific; J20605-AP) for 1 h at room temperature. Primary antibodies were incubated in LICOR Antibody Diluent (LICOR; 927–65001) or CanGet Signal Immunoreaction Enhancer Solution 1 (TOYOBO; NKB-101) overnight at 4°C. After three washes in TBST, the blots were incubated in LICOR Antiibody Diluent (LICOR; 927–65001) or CanGet Signal Immunoreaction Enhancer Solution 2 (TOYOBO; NKB-201) for 1 h at room temperature. When required, the blots were stripped in Antibody Stripping Buffer (Thermo Scientific; 46430). Protein blots were visualized and imaged using LICOR Odyssey system.

#### Generation of LentiCRISPR V2 vectors and viral production

CRISPR small-guide (sg)RNA sequences for targeting *SF3B1*, *Sf3b1*, *Sf3b2*, *Sf3b3*, *Sf3b4*, *Sf3b5*, *Sf3b14*, *Phf5a*, and *Zbp1* were designed using publicly available Broad Institute CRISPick small-guide RNA designer, https://portals.broadinstitute.org/gppx/crispick/public. sgRNA sequences are cloned into lentiCRISPR V2 vector (Addgene; plasmid #. 52961). Plasmid sequences containing the sgRNA sequences were validated by Sanger sequencing through GENEWIZ. The following target sequences were used:

sg*SF3B1* forward3: CACCGGAGAACTAAAAGTCGTCAA

sg*SF3B1* reverse3: AAACTTGACGACTTTTAGTTCTCC

sg*SF3B1* forward4: CACCGATAGCGGTTCAATGACCACG

sg*SF3B1* reverse4: AAACCGTGGTCATTGAACCGCTATC

sg*Sf3b1* forward3: CACCGAGACTGAAATTCTCGAATG

sg*Sf3b1* reverse3: AAACCATTCGAGAATTTCAGTCTC

sg*Sf3b1* forward4: CACCGATTACTATGCTAGAGTGGA

sg*Sf3b1* reverse4: AAACTCCACTCTAGCATAGTAATC

sg*Sf3b2* forward1: CACCGAAGTACCTTCAAGGCAAACG

sg*Sf3b2* reverse1: AAACCGTTTGCCTTGAAGGTACTTC

sg*Sf3b2* forward2: CACCGTCGTCCTATAGGGAGTCGCG

sg*Sf3b2* reverse2: AAACCGCGACTCCCTATAGGACGAC

sg*Sf3b3* forward1: CACCGAACACTCTGGTGTATCACG

sg*Sf3b3* reverse1: AAACCGTGATACACCAGAGTGTTC

sg*Sf3b3* forward2: CACCGATGGCGTTTAGGCTAACAGG

sg*Sf3b3* reverse2: AAACCCTGTTAGCCTAAACGCCATC

sg*Sf3b4* forward1: CACCGGTCAACACCCACATGCCCA

sg*Sf3b4* reverse1: AAACTGGGCATGTGGGTGTTGACC

sg*Sf3b4* forward2: CACCGAACTCCAAGCTGTACCTGG

sg*Sf3b4* reverse2: AAACCCAGGTACAGCTTGGAGTTC

sg*Sf3b5* forward1: CACCGCCACGCCGACACCACCAAG

sg*Sf3b5* reverse1: AAACCTTGGTGGTGTCGGCGTGGC

sg*Sf3b5* forward2: CACCGACTCCTACTGCTCCTACAT

sg*Sf3b5* reverse2: AAACATGTAGGAGCAGTAGGAGTC

sg*Sf3b14* forward1: CACCGAAACGTTGAATCCTGATAGG

sg*Sf3b14* reverse1: AAACCCTATCAGGATTCAACGTTTC

sg*Sf3b14* forward2: CACCGAATCCTGATAGGTGGTCGC

sg*Sf3b14* reverse2: AAACGCGACCACCTATCAGGATTC

sg*Phf5a* forward1: CACCGCAGGGTGCAGGGACGCACGT

sg*Phf5a* reverse1: AAACACGTGCGTCCCTGCACCCTGC

sg*Phf5a* forward2: CACCGCTCATCACATATGCGGACCA

sg*Phf5a* reverse2: AAACTGGTCCGCATATGTGATGAGC

sg*Zbp1* forward1: CACCGTGAGCTATGACGGACAGACG

sg*Zbp1* reverse1: AAACCGTCTGTCCGTCATAGCTCAC

sg*Zbp1* forward2: CACCGCTTCCCTGCATCCCCCACG

sg*Zbp1* reverse2: AAACCGTGGGGGATGCAGGGAAGC

sg*Zbp1* forward3: CACCGCAGGTGTTGAGCGATGACGG

sg*Zbp1* reverse3: AAACCCGTCATCGCTCAACACCTGC

sg*Zbp1* forward4: CACCGTGTGCTGACAAATAATCGCA

sg*Zbp1* reverse4: AAACTGCGATTATTTGTCAGCACAC.

To produce lentiviruses, HEK293 cells were seeded at approximately ~40–50% confluence the day before transfection in 60mm dishes. Per dish 1 μg of pMD2.G (Addgene; plasmid #. 12259), 1 μg of psPAX2 (Addgene; plasmid #. 12260), and 1 μg of target plasmid were transfected using X-tremeGene 9 Transfection Reagent (Roche; XTG9-RO) according to the manufacturer’s instructions. The viral supernatant produced by transfected HEK293T were combined (collected at 48 h and 72 h post transfection) and filtered through 0.45 μm filters with a syringe.

#### Genetic deletion by CRISPR-Cas9

For *SF3B1* ablation, human cell lines are cultured with supernatant containing viral particles produced by HEK293T supplemented with 8 μg/mL polybrene (Santa Cruz; sc-134220) and centrifuged for 2 h at 2000 rpm. For *Sf3b1*, *Sf3b2*, *Sf3b3*, *Sf3b4*, *Sf3b5*, *Sf3b14*, *Phf5a*, and *Zbp1* gene deletion, mouse cell lines were cultured with supernatant containing viral particles produced by HEK293T supplemented with 8 μg/mL polybrene (Santa Cruz; sc-134220) and centrifuged for 2 h at 2000 rpm. All cells were cultured with supernatant containing viral particles for overnight at 37°C in 5% CO_2_ humidified incubator, followed by a complete media change and allowed to grow for 48 h prior to antibiotic selection. Infected cells were selected with media supplemented with 1 μg/mL puromycin (Gibco; A11138–03) for 72 h prior to experimentation.

All CRISPR-Cas9 mediated knockdown experiments in this publication used pooled cell populations immediately after antibiotic selection, unless otherwise stated.

#### IncuCyte real time cytotoxicity assays

To capture and quantify real time Pladienolide B (PlaB) cytotoxicity in human and mouse cell lines, cells were seeded in 96-well plate and allowed to adhere prior to experimentation. Followed by a complete media change containing IncuCyte Cytotox Green Dye for Counting Dead Cells (Sartorisu; 4633) according to the manufacturer’s instructions. Real time imaging of cell fluorescence and data analyses were performed using IncuCyte Live-Cell Analysis System SX5. Data were exported to excel format using the IncuCyte Software (Sartorius), and data were plotted using PRISM V10 (GraphPad).

#### siRNA transfection

Silencer Select SF3B1 siRNA (Thermo Fisher; 4392420; s223598) and Silencer Select Negative Control 1 (Thermo Fisher; 4390844) were purchase from Thermo Fisher Scientific and used for siRNA silencing experiments in both human and mouse cell lines. Cells were plated on 6-well plates and allowed to adhere overnight to reach a confluence of 30%–50% and then transfected with Lipofectamine RNAiMAX Reagent (Thermo Fisher; 13778–150) for 72 h according to the manufacturer’s instructions. When applicable, cell lysates were collected for immunoblotting procedures.

#### Lung tumors and cancer cell line encyclopedia (CCLE) necroptosis gene signature analyses

mRNA sequencing data were obtained from SCLC patient tumor samples at the European Genome-Phenome Archive under the accession code EGAS00001000925^65^ and human lung tumor cell lines from CCLE and analyzed based on ZBP1, RIPK3, and MLKL necroptosis pathway gene signatures.

#### RNA immunoprecipitation and sequencing (RIP-Seq)

*Zbp1*^−/−^ MEF EV cells were treated with DMSO or 50 nM PlaB for 18 h, and then the cells were collected on ice and RNA immunoprecipitation (RIP) was conducted using a Magna RIP RNA-Binding Protein Immunoprecipitation Kit (EMD Millipore; 17–700) following the manufacturer’s instructions. In brief, MEF EV cells were lysed in ice-cold RIP lysis buffer followed by incubation with RIP buffer containing magnetic beads conjugated with the Z-Nucleic Acid antibody (Absolute Antibody; Ab00783.23.0) and the isotype control antibody (provided in the kit) overnight at 4°C with gentle rotation. Sample were then incubated with proteinase K (provided in the kit), and immunoprecipitated RNAs were recovered by phenol:chloroform:isoamyl alcohol purification procedure. Recovered RNAs were assessed for quality using 4200 TapeStation System High Sensitivity RNA ScreenTape Analysis (Agilent; 5067–5579, 5067–5580, 5067–5581) and 2100 Bioanalyzer RNA 6000 Nano Assay (Agilent; 5067–1513. 5067–1514, 5067–1535). RNAs were further quantified using a QuantFluor RNA System (Promega; E3310) before library preparation. Ribosomal RNA (rRNA) was removed using RiboMinus Core Module V2 (Thermo Fisher; A15015) and RiboMinus Concentration Module (Thermo Fisher; K155005) following the manufacturer’s instructions.

RNA fragmentation, cDNA reverse transcription, and Illumina sequencing library preparations were performed by the Novogene RIP-Seq services using the NEB Next Ultra II RNA Library Prep Kit for Illumina (NEB; E7770L) following the manufacturer’s instructions. Libraries were analyzed for fragment distributions using the 2100 Bioanalyzer RNA 6000 Nano Assay (Agilent) and quantified using the Qubit 2.0 Fluorometer (Thermo Fisher). Followed by Illumina paired-end 150 (PE150) cycle sequencing on a NovaSeq 6000 (Illumina). Raw data were downloaded for RIP-Seq analyses.

#### RIP-qRT-PCR

RNAs from the RIP samples were reverse transcribed into cDNA using SuperScript IV VILO master mix (Thermo Fisher; 11756050) according to the manufacturer’s instructions. Followed by qPCR using the POWER SYBR Green (Applied Biosystems; 4367659) and the results were analyzed by the Applied Biosystem QuantStudio 6 Pro. Relative gene expression were normalized to input using the -ΔΔCt method. The following primers were used:

*mArc* forward: CACAGGAAGCAGCAAGATGGTG

*mArc* reverse: CCTATCCTGACCAAGCCTCAG

*mCactin* forward: GGCTTTCACAGGGCCTTGGCA

*mCactin* reverse: AGGAGGGTGTCACTATCTCAGTC

*mChpf* forward: CAGGGGAAAAAGGGGCCATGAGC

*mChpf* reverse: CTGGGCTTAGGGAGTCAAGGGA

*mCluh* forward: CCTCTGCCACCATGCCCTGC

*mCluh* reverse: TGTGGCAGGCAGGGGAATGGA

*mPcyt2* forward: CTAGAGGTGACACGCACACAGAA

*mPcyt2* reverse: CAAACCCTGTTTATGAGCCTAGTG

*mActb* forward: GGCTGTATTCCCCTCCATCG

*mActb* reverse: CCAGTTGGTAACAATGCCATGT

*mGapdh* forward: TGACCTCAACTACATGGTCTACA

*mGapdh* reverse: CTTCCCATTCTCGGCCTTG.

#### Enzymatic probing of double-stranded Z-RNA structure

MEF EV cells were treated with DMSO or 50 nM Pladienolide B (Cayman Chemical; 16538) for 18 h, cell pellets were collected in biological triplicates in ice-cold PBS and then pelleted briefly at 4°C for 5 min at 1500 rpm. Cells were lysed in 1mL RIP buffer (25 mM HEPES pH 7.2, 150 nM NaCl, 5 mM MgCl2, 0.1% Igepal CA-630, 1 U/uL RNasin Plus) for 5 min on ice and then transferred to −80°C for at least overnight. The supernatant was transferred to a new Eppendorf tube, and the cell lysates were treated with or without 5U RNase One (Promega, M4261) for 30 min at room temperature. Sample RNA supernatants were extracted using Trizol at a ratio of 1 volume RIP buffer to 5 volumes of Trizol reagent (Thermo Fisher; 15596018) and chloroform was added at a ratio of 1:5. Aqueous layer was collected and then transferred to the RNA Clean and Concentrate columns (Zymo; R1017) and isolated following the manufacturer’s instructions. First-stranded cDNA synthesis was performed with SuperScript III (Thermofisher Scientific; 18080–044) with the following modifications: RNA was heated to 70C for 5 min to reduce secondary structure, followed by reverse transcription according to the manufacturer’s instruction. Transcript abundance was then measured using RT-qPCR with the POWER SYBR Green (Applied Biosystems; 4367659) using 20ng of input cDNA, assuming 1:1 RNA to cDNA synthesis. Relative transcript abundance in samples treated with and without RNase One was normalized to *Actb* and assessed using the Applied Biosystem QuantStudio 6 Pro. The dsRNA/ssRNA fold enrichment was then calculated using the ΔΔCt method comparing the relative transcript abundance in the samples treated with RNase One to the samples treated without RNase One that was previously published.^18^ The relative abundance was calculated between PlaB-treated over DMSO-treated conditions. *Gapdh*, which is not expected to form dsRNA under normal conditions, was used as a control for non-dsRNA forming transcripts in the two experimental conditions tested, PlaB and DMSO. All samples were normalized to *ActB*.

#### Double-stranded Z-RNA structure prediction

Double-stranded RNA prediction was done using the publicly available RNAfold tool at: http://rna.tbi.univie.ac.at/cgi-bin/RNAWebSuite/RNAfold.cgi using the default settings.

#### Generation of *Zbp1* knockout (KO) RPP-A single cell clones

RPP-A mouse small cell lung cancer was infected with sg*Zbp1* lentiviral constructs. After three days of antibiotic selection, cells then were plated on 96 wells at a limiting dilution of 1 cell per 100 μL per well in complete growth media containing antibiotics. Wells containing single cell colonies were selected and passaged, cells lysates were collected and validated by western blot by the absence of Zbp1 protein. Single clone RPP-A *Zbp1 KO* cells were then used for experiments in Figures 4F, 4G, and 5N.

#### CellTiter-Glo Luminescent cell viability assay

Cells were seeded and treated with DMSO and pladienolide B in 96 well plate in triplicates (Corning; 3610) for 18 h. Reconstituted lyophilized enzyme and substrate mixture was allowed to equilibrate to room temperature, and the cell and CellTiter Glo enzyme/substrate mixture was mixed according to the manufacturer’s instructions (Promega; G7571). The plate was incubated at room temperature for 10 min after homogenization and measured with CLARIOstar^Plus^ (BMG LABTECH).

#### GEMM-derived murine SCLC model

Isogenic RPP and RPP-A SCLC murine cell lines were established by Dr. Matthew G. Oser (Dana-Farber Cancer Institute, Boston, MA, USA), and were derived from triple knockout of *Trp53*, *Rb1*, and *Rb2* genes using AAV vectors encoding Cre-recombinase and sgRNAs in LSL-Cas9 C578BL/6 mice.

#### *In vivo* tumor studies and treatments

For tumor burden and immunotherapeutic experiments, 8.0 × 10^6^ RPP cells were subcutaneously implanted into the right flank of the six-to-eight weeks old male and female C57BL/6J *Zbp1*^−/−^ and *Zbp1*^+/+^ mice with cell suspension in 1:1 ratio with Matrigel (Corning; 354234). For all *in vivo* experiments, tumors were measured every 2–3 days once palpable tumors were observed using a digital caliper. For all *in vivo* experiments, mice were randomized onto different experimental groups when the tumors reached 150–300 mm^3^ based on the formula: tumor volume = (length × width^2^)/2.^91–93^ DMSO and Pladienolide B treatments were given intratumorally, and immunotherapy treatments (IgG isotype control (BioXCell; BP0089), and αPD1 (BioXCell; BP0146) were given intraperitoneally (i.p.) at a dose of 200 μg per cycle. For combination treatments, DMSO treatment and Pladienolide B treatment in combination with IgG or αPD1 were given simultaneously for four doses at an interval every 2–3 days. Tumors were harvested when a single mouse’s tumor size from any group exceeded 2000 mm^3^. For survival studies, mice were monitored for endpoint until any of given criteria was met: tumor size exceeded 2000 mm^3^, weigh loss greater than 20%, tumor ulceration, tumor size exceeded 10% body weight, decreased activity, and infections.

#### Single cell suspension of RPP tumors with enzymatic dissociation

Primary tumor samples were harvested and placed into a 100 mm Petri dish with ice-cold PBS. Tumors were quickly minced with a sterile scalpel into 3 mm–4 mm fragments and pelleted briefly at 4°C for 5 min at 1500 rpm. Tumor pellets were enzymatically digested with 2 collagenase solution supplemented with 2 mg/mL Collagenase (Sigma, C5138), 2 μg/mL DNase I (Roche, 10104159001), and 1 tablet of EDTA-free protease inhibitor cocktail per 50mL solution (Roche, 11836170001). Tumor suspensions were incubated at 37°C with agitation until no visible tumor chunks are visible. Tumor suspensions were washed with complete RPMI growth medium supplemented with 10% FBS and 1% Penicillin-Streptomycin and passed through 100 μM cell strainers. Tumor pellets were washed twice with PBS and briefly pelleted at 4°C for 5 min. Single cell tumor suspensions were subsequently used for flow cytometry immune profiling with the following antibodies: PI PE CF594 (Sigma; P4170), CD45 BV510 (BioLegend; 103138), NK1.1 PE (BioLegend; 108708), CD3 PE/Cy7 (BioLegend; 100220), CD4 BV421 (BioLegend; 116023), CD8a APC/Cy7 (BioLegend; 100714), TCR γδ PerCp Cy5.5 (BioLegend; 118118), CD11b APC (BioLegend; 101212), F4/80 Alexa Fluor 700 (BioLegend; 123130).

#### Isolation of primary lung fibroblasts

Primary mouse lung fibroblasts were isolated from six-to-eight-weeks old *Zbp1*^+/+^ and *Zbp1*^−/−^ mice lung tissues. Mouse lung tissues were quickly dissected and placed on ice-cold PBS. Lung tissues were cut into 1mm–2mm pieces with sterile scalpel and then were digested enzymatically with the digestion buffer containing high glucose DMEM supplemented with 1% FBS, 1% Penicillin-Streptomycin, 10 μg/mL of DNase I (Sigma; 10104159001), and 0.4 mg/mL of collagenase P (Roche; 11213865001). Minced lung tissues were incubated at 37°C incubator with gentle agitation for 30 min. After the incubation period, enzymatic digestion was stopped by adding the wash buffer (high glucose DMEM with 1% FBS and 1% Penicillin-Streptomycin) and cell were briefly centrifuged at 4°C for 10 min at 300g. Pellets were resuspended in 0.25% Trypsin-EDTA for 20 min at 37°C with gentle agitation. Tissue suspensions were washed with the wash buffer and pelleted briefly at 4°C for 5 min. Primary mouse lung fibroblasts were then resuspended in complete growth medium (high glucose DMEM supplemented with 10% FBS and 1% Penicillin-Streptomycin and cultured at 37°C with 5% CO_2_. Passages 3 primary mouse lung fibroblasts were used for experiments.

#### RIP-seq analyses

##### Quality control (QC), data processing, and alignment

RIP-Seq sequencing FASTQ files were processed using Nextflow nf-core/RNAseq pipeline (v3.11) framework.^82^ Raw sequencing reads were quality-checked using FastQC^83^ and the adapter sequences were trimmed by Trim Galore. Adapters and low quality of reads were excluded for down streaming analysis. The trimmed reads were aligned to the mouse reference genome (GRCm38) using the STAR aligner.^84^ Picard MarkDuplicates tool was used to filter and remove duplicate reads. Reads that failed to map properly to the genome assembly were excluded from further analyses. The alignment files were processed and transcript gene expression levels were quantified using Salmon.^85^ This produced raw counts and normalized expression values for downstream analyses.

##### RIP-seq gene enrichment analysis

Gene differential expression analysis was performed using DESeq2 (v 1.44) R package^86^ for input controls, Z22-enriched genes. Of note, input RNA samples (PlaB- and DMSO-treated) were not subjected to immunoprecipitation and serve as controls for normalization and comparisons. Two biological replicate gene expression values were compared between PlaB Z22-immunoprecipitated RNA samples over PlaB 10% input controls versus DMSO Z22-immunoprecipitated RNA samples over DMSO 10% input controls. Enrichment of Z-RNA species were considered enriched when the Z22-immunoprecipitated/10% input ratio exhibited at least 2-fold increase (log2 fold-change ≥1) and with an adjusted *p*-value (FDR) < 0.05.

##### RIP-seq intron retention analysis

Intron retention (IR) events quantification was analyzed using iREAD (version 0.8.9). Aligned BAM files generated from STAR Alignment were used as the inputs for iREAD.^87^ iREAD calculated IR ratios, FPKM values, and read counts for each annotated and independent intron across all the samples. Independent IRs are introns that do not have any overlap with exons of splicing isoforms or gene. To rigorously demonstrate PlaB-treated Z22-specific enrichment of intron-containing transcripts in the RIP-Seq dataset, we performed a two-step differential intron retention analysis. First, we applied the lmFit function from the limma package^88^ to log_2_(FPKM+1)-transformed expression values of intronic regions, followed by the eBayes (edgeR package)^89^ method to assess statistical significance with FDR <0.25 and log_2_ fold-change ≥1 as the cutoff. Intron retention events were considered significantly increase in PlaB Z22 vs. PlaB input but not significantly changed in DMSO Z22 vs. DMSO input. These filtering ensures that the identified intron retention events reflect Z22-specific binding when spliceosome is inhibited rather than the background effects or global transcriptomic changes caused by PlaB.

##### RIP-seq data visualization

Differential expression and intron retention events were visualized using R packages (ggplot2) and Morpheus for clustering analysis.

### QUANTIFICATION AND STATISTICAL ANALYSIS

Statistical analyses are described in each figure legend. All data are plotted as means, with error bars representing the S.E.M. unless stated otherwise. Data shown are representative of at least three independent biological replicates, unless otherwise indicated. Statistical analyses were performed using Prism V10 (GraphPad Software). For all quantitative measurements, normal distribution was assumed, with two-sided unpaired *t*-tests unless otherwise stated. Data were analyzed by Student’s t test between two groups, two-way ANOVA, or Mann-Whitney U test as appropriate. All figures and analyses were generated using Prism V10 (GraphPad Software) or R.

## Supplementary Material

Supplementary Figures (Figures S1-S7)

Table S1. List of differentially expressed pre-mRNAs in Z-conformation identified by Z22-antibody pull-down after PlaB treatment.

Table S2. List of all statistically significant intron retention events in PlaB treatment compared to DMSO control.

Table S3. List of 35 significantly enriched intron retention events for Morpheus clustering analysis.

Table S4. List of all significantly enriched intron retention events for Morpheus clustering analysis.

Supplemental information can be found online at https://doi.org/10.1016/j.celrep.2025.116384.

## Figures and Tables

**Table:** KEY RESOURCES TABLE

REAGENT or RESOURCE	SOURCE	IDENTIFIER
Antibodies		
Anti-Z-DNA/Z-RNA (Z22)	Absolute Antibody	Cat# Ab00783-23.0; RRID: AB_3065276
Anti-MLKL (phospho S345)	ABCAM	Cat# ab196436; RRID: AB_2687465
NK1.1 Monoclonal Antibody (PK136)	Thermo Fisher Scientific	Cat# MA1-70100; RRID: AB_2296673
PerCP/Cyanine5.5 anti-mouse TCR γ/δ Antibody	BioLegend	Cat# 118118; RRID: AB_10612756
CD140a (PDGFRA) Monoclonal Antibody	Thermo Fisher Scientific	Cat# 14-1401-82; RRID: AB_467491
Donkey anti-Rabbit IgG, Alexa Fluor 488	Thermo Fisher Scientific	Cat# A32790; RRID: AB_2762833
Donkey anti-Rabbit IgG, Alexa Fluor 594	Thermo Fisher Scientific	Cat# A32754; RRID: AB_2762827
IRDye 680RD Goat anti-Rabbit	LI-COR	Cat# 926-68071; RRID: AB_10956166
IRDye 800CW Goat anti-Mouse	LI-COR	Cat# 926-32210; RRID: AB_621842
Zombie Green Fixable Viability Kit	BioLegend	Cat# 423112
Anti-MLKL (phosphor S345) antibody [EPR9515(2))	Abcam	Cat# AB196436; RRID: AB_2687465
Anti-MLKL (phosphor S358) antibody [EPR9514]	Abcam	Cat# ab187091; RRID: AB_2619685
RIP3 Antibody	ProSci	Cat# 2283; RRID: AB_203256
Anti-ZBP1, mAb (Zippy-1)	AdipoGen Life Sciences	Cat# AG-20B-0010-C100; RRID: AB_2490191
MLKL (D216N) Rabbit mAb	Cell Signaling Technology	Cat# 14993S; RRID: AB_2721822
SF3B1 (D7L5T) Rabbit mAb	Cell Signaling Technology	Cat# 14434S; RRID: AB_2798479
SF3B2 Polyclonal Antibody	Thermo Fisher Scientific	Cat# PA5-96456; RRID: AB_2808258
SF3B3 Polyclonal Antibody	Proteintech	Cat# 14577-1-AP; RRID: AB_2270189
SF3B4 Polyclonal Antibody	Thermo Fisher Scientific	Cat# PA5-101909; RRID: AB_2851341
SF3B5 Polyclonal Antibody	Proteintech	Cat# 15525-1-AP; RRID: AB_10596628
SF3B14 Polyclonal Antibody	Proteintech	Cat# 12379-1-AP; RRID: AB_2186517
PHF5A Polyclonal Antibody	Proteintech	Cat# 15554-1-AP; RRID: AB_2165365
β-Actin (8H10D10) Mouse mAb	Cell Signaling Technology	Cat# 3700S; RRID: AB_2242334
β-Actin (13E5) Rabbit mAb	Cell Signaling Technology	Cat# 4970S; RRID: AB_2223172
Brilliant Violet 510 anti-mouse CD45 Antibody	BioLegend	Cat# 103138; RRID: AB_2563061
PE/Cyanine7 anti-mouse CD3 Antibody	BioLegend	Cat# 100220; RRID: AB_1732057
APC/Cyanine7 anti-mouse CD8a Antibody	BioLegend	Cat# 100714; RRID: AB_312753
Brilliant Violet 421 anti-mouse CD4 Antibody	BioLegend	Cat# 116023; RRID: AB_2800579
PE anti-mouse NK-1.1 Antibody	BioLegend	Cat# 108708; RRID: AB_313395
APC anti-mouse/human CD11b Antibody	BioLegend	Cat# 101212; RRID: AB_312795
Alexa Fluor 700 anti-mouse F4/80 Antibody	BioLegend	Cat# 123130; RRID: AB_2293450
PerCP/Cyanine5.5 anti-mouse TCR γ/δ Antibody	BioLegend	Cat# 118118; RRID: AB_10612756
Bacterial and virus strains		
NEB Stable Competent *E. coli* (High Efficiency)	New England Biolabs (NEB)	Cat# C3040I
Biological samples		
Primary wild-type (WT) mouse lung fibroblasts (*Zbp1*^−/−^)	This paper	N/A
Primary Zbp1-knockout (KO) mouse lung fibroblasts (*Zbp1*^+/+^)	This paper	N/A
Chemicals, peptides, and recombinant proteins		
Pladienolide B	Cayman Chemicals	Cat# 445493-23-2; ID 16538
Matrigel Matrix	Corning	Cat# 354234
Bovine Serum Albumin (BSA)	Fisher Scientific	Cat# BP9703-100
RNaseA	Thermo Fisher Scientific	Cat# EN0531
RNaseH	Thermo Fisher Scientific	Cat# 18021014
DNaseI Set	Zymo Research	Cat# E1011-A
Glycogen	Thermo Fisher Scientific	Cat# R0551
ITS Supplement	Sigma	Cat# 11884
B-Estradiol	Sigma	Cat# E2257
Hydrocortisone	Sigma	Cat# H4001
FBS	Hyclone	Cat# AAF204954
RPMI 1640 Medium	Corning	Cat# MT-50-020-PB
Sodium Pyruvate	Sigma	Cat# P2256
HEPES	Fisher Scientific	Cat# BP310-500
Dextrose (D-) Glucose	Fisher Scientific	Cat# D15-500
Dulbecco’s Modification of Eagle’s Medium (DMEM)	Corning	Cat# MT50-013-PB
Phosphate Buffered Saline 10X	Corning	Cat# 46-013-CM
Penicillin Streptomycin	Corning	Cat# 30-002-CI
L-Glutamine	Corning	Cat# 25-005-CI
Paraformaldehyde, 4% in PBS	Thermo Fisher Scientific	Cat# J61899
Phosphate Buffered Saline	Sigma	Cat# D5652
Triton X-100	Sigma	Cat# X100-500ML
MAXblock Blocking Medium	Active Motif	Cat# 102224
ProLong Gold Antifade Mountant with DNA Stain DAPI	Thermo Fisher Scientific	Cat# P36935
Sucrose	Thermo Fisher Scientific	Cat# 036508.A1
Pierce RIPA Buffer	Thermo Fisher Scientific	Cat# 89900
Halt Protease Inhibitor Cocktail (100X)	Thermo Fisher Scientific	Cat# 78430
Halt Phosphatase Inhibitor Cocktail (100X)	Thermo Fisher Scientific	Cat# 78428
Chameleon Duo Pre-Stained Protein Ladder	LICORbio	Cat# 928-60000
Puromycin	GIBCO	Cat# A11138-03
Can Get Signal Immunoreaction Enhancer Solution 1	TOYOBO	Cat# NKB-101
Can Get Signal Immunoreaction Enhancer Solution 2	TOYOBO	Cat# NKB-201
Intercept Antibody Diluent	LI-COR	Cat# 927-65001
CoverGrip Coverslip Sealant	Biotium	Cat# 23005
Tween 20	Thermo Fisher Scientific	Cat# J20605-AP
IGEPAL	Sigma	Cat# 9002-93-1
1M MgCl_2_	Invitrogen	Cat# AM9530G
0.5M EDTA Solution (100X)	Thermo Fisher Scientific	Cat# 1860851
5M NaCl	Thermo Fisher Scientific	Cat# AM9759
Ambion DEPC-Treated Water	Thermo Fisher Scientific	Cat# AM9922
SuperScript III Reverse Transcriptase	Thermo Fisher Scientifics	Cat# 18080-044
RNaseOUT Ribonuclease Inhibitor	Thermo Fisher Scientific	Cat# 10777019
Power SYBR Green PCR Master Mix	Applied Biosystems	Cat# 4367659
Oligo (dT) 12–18 Primer	Life Technologies	Cat# 18418012
Random Primers	Thermo Fisher Scientific	Cat# 48190011
dNTP Mix, 10mM each	Thermo Fisher Scientific	Cat# R0191
TRIzol Reagent	Thermo Fisher Scientific	Cat# 15596018
2-Mercaptoethanol	Bio-Rad	Cat# 1610710
cOmplete Mini, EDTA-free	Roche	Cat# 11836170001
Collagenase P	Roche	Cat# 11213865001
Restore PLUS Western Blot Stripping Buffer	Thermo Fisher Scientific	Cat# 46430
*InVivo*Pure pH 7.0 Dilution Buffer	BioXCell	Cat# IP0070
*InVivo*Plure pH 6.5 Dilution Buffer	BioXCell	Cat# IP0065
*InVivo*Plus rat IgG2a isotype control, Clone 2A3	BioXCell	Cat# BP0089
*InVivo*Plus anti-mouse PD-1 (CD279), clone RMP1-14	BioXCell	Cat# BP0146
SuperScript IV VILO Master Mix	Thermo Fisher Scientific	Cat# 11756050
RNasin Plus RNase Inhibitor	Promega	Cat# N2615
RNase ONE Ribonuclease	Promega	Cat #M4261
GSK843	Sigma	Cat# SML2001-5MG
Z-VAD-FMK	Sellectchem	Cat# S7023
Necrostatin-1	APExBio	Cat# A4213
Cycloheximide	Sigma	Cat# C4859-1ML
Recombinant Mouse TNF alpha (aa 80–235) Protein	R&D Systems	Cat# 410-MT
Collagenase	Sigma	Cat# C5138-1G
DnaseI from bovine pancreas grade II	Roche	Cat# 10104159001
MicroAmp Fast Optical 96-Well Reaction Plate with Barcode	Applied Biosystems	Cat# 4346906
Propidium Iodide	Sigma	Cat# P4170
10X TBS	Bio-Rad	Cat# 1706435
Mini Trans-Blot Filter Paper	Bio-Rad	Cat# 1703932
Phosphate Buffered Saline 10X Molecular Biology Grade	Corning	Cat# 46-013-CM
RBC Lysis Buffer (10X)	BioLegend	Cat# 420302
Chloroform	Fisher Scientific	Cat# BP1145-1
Phenol:Chloroform:Isoamyl Alcohol 125:24:1	Sigma	Cat# P1944-100ML
Nitrocellulose Membrane, 0.45μM	Bio-Rad	Cat# 1620115
Nunc Lab-Tek II Chamber Slide System, 8 Wells	Thermo Fisher Scientific	Cat# 154941
Cover Slips Super Slip	Epredia	Cat# 12450S
Polybrene (CAS 28728-55-4)	Santa Cruz	Cat# sc-134220A
Sodium Bicarbonate	Fisher Scientifics	Cat# BP328-500
Actinomycin D	Sigma	Cat# A9415
Critical commercial assays		
Pierce BCA Protein Assay Kit	Thermo Fisher Scientific	Cat# 23225
CellTiter-Glo Luminescent Cell Viability	Promega	Cat# G7571
Bio-Rad Wet Tank Blotting System	Bio-Rad	Cat# 1658034
RNA Clean & Concentrator – 25	Zymo Research	Cat# R1017
RNA Binding Buffer	Zymo Research	Cat# R1013-2-100
RNA Wash Buffer	Zymo Research	Cat# R1003-3-48
Magna RIP Kit	EMD Millipore	Cat# 17-700
High Sensitivity RNA ScreenTape	Agilent Technologies	Cat# 5067-5579
High Sensitivity RNA ScreenTape Ladder	Agilent Technologies	Cat# 5067-5581
High Sensitivity RNA ScreenTape Sample Buffer	Agilent Technologies	Cat# 5067-5580
Agilent RNA 6000 Pico Kit	Agilent Technologies	Cat# 5067-1513
Agilent RNA 5000 Pico Ladder	Agilent Technologies	Cat# 5067-1515
Agilent RNA 6000 Pico Reagents	Agilent Technologies	Cat# 5067-1514
QuantiFluor RNA System	Promega	Cat# E3310
NEBNext Ultra II RNA Library	New England Biolabs	Cat# E7770L
RiboMinus Core Module v2	Life Technologies	Cat# A15015
RiboMinus Eukaryote Oligo Moldule v2	Life Technologies	Cat# A15017
RiboMinus Concentration Module	Life Technologies	Cat# K155005
Lipofectamine RNAiMAX Reagent	Thermo Fisher Scientific	Cat# 13778-150
IncuCyte Cytotox Green Reagent	Sartorius	Cat# 4633
X-tremeGENE 9 DNA Transfecton Reagent	Roche	Cat# XTG9-RO
Deposited data		
RIP-Sequencing Raw DataSets for EV MEFs treated with DMSO vs. PlaB	This Study	GEO: GSE307540
Experimental models: Cell lines		
NCI-H446	ATCC	Cat# HTB-171
RPP (mSCLC Cell Line, suspension)	Provided by Dr. Mathew Oser (Dana-Farber Cancer Institute)	N/A
RPP-A (mSCLC Cell line, adherent)	Provided by Dr. Mathew Oser (Dana-Farber Cancer Institute)	N/A
Immortalized Wild-type (WT) mouse embryonic fibroblasts (MEFs)	Provided by Dr. Balachandran Siddharth (Fox Chase Cancer Center)	N/A
Immortalized Empty Vector (EV) MEFs	Provided by Dr. Balachandran Siddharth (Fox Chase Cancer Center)	N/A
Immortalized Flag-ZBP1 MEFs	Provided by Dr. Balachandran Siddharth (Fox Chase Cancer Center)	N/A
Immortalized ΔZα MEFs	Provided by Dr. Balachandran Siddharth (Fox Chase Cancer Center)	N/A
Human pancreatic cancer-associated fibroblasts (CAFs)	Provided by Dr. Edna Cukierman (Fox Chase Cancer Center)	N/A
Human lung CAFs	Provided by Dr. Edna Cukierman (Fox Chase Cancer Center)	N/A
RPP-A EV	This paper	N/A
RPP-A sg*Zbp1* #1 (single cell clone)	This paper	N/A
RPP-A sg*Zbp1* #2 (single cell clone)	This paper	N/A
RPP-A sg*Zbp1* #3 (single cell clone)	This paper	N/A
RPP-A sg*Zbp1* #4 (single cell clone)	This paper	N/A
Experimental models: Organisms/strains		
C57BL/6J	The Jackson Laboratory	Cat# 000664; RRID: IMSR_JAX:000664
*Zbp1*^−/−^ Mice	Provided by Dr. Balachandran Siddharth (Fox Chase Cancer Center)	N/A
*Zbp1*^+/+^ Mice	Provided by Dr. Balachandran Siddharth (Fox Chase Cancer Center)	N/A
Oligonucleotides		
Silencer Select Negative Control #1 siRNA	Thermo Fisher Scientific	Cat# 4390844
SF3B1 Silencer Select Pre-designed siRNA	Thermo Fisher Scientific	Cat# 4392420; ID: s223598
LRDD Silencer Select Pre-designed siRNA	Thermo Fisher Scientific	Cat# 4392420; ID: s30843
PTEN Silencer Select Pre-designed siRNA	Thermo Fisher Scientific	Cat# 4427037; ID: s61224
Pbrm1 Silencer Select Pre-designed siRNA	Thermo Fisher Scientific	Cat# 4390771; ID: s232536
RBM5 Silencer Select Pre-designed siRNA	Thermo Fisher Scientific	Cat# 4427037; ID: s19842
Herc4 Silencer Select Pre-designed siRNA	Thermo Fisher Scientific	Cat# 4390771; ID: s84900
Ddx31 Silencer Select Pre-designed siRNA	Thermo Fisher Scientific	Cat# 4390771; ID: s105733
Hdac6 Silencer Select Pre-designed siRNA	Thermo Fisher Scientific	Cat# 4390771; ID: s67426
Hhx32 Silencer Select Pre-designed siRNA	Thermo Fisher Scientific	Cat# 4390771; ID: s97751
STK38 Silencer Select Pre-designed siRNA	Thermo Fisher Scientific	Cat# 4427037; ID: s22337
sg*SF3B1* forward3: CACCGGAGAACTAAAAGTCGTCAA	This Paper	N/A
sg*SF3B1* reverse3: AAACTTGACGACTTTTAGTTCTCC	This Paper	N/A
sg*SF3B1* forward4: CACCGATAGCGGTTCAATGACCACG	This Paper	N/A
sg*SF3B1* reverse4: AAACCGTGGTCATTGAACCGCTATC	This Paper	N/A
sg*Sf3b1* forward3: CACCGAGACTGAAATTCTCGAATG	This Paper	N/A
sg*Sf3b1* reverse3: AAACCATTCGAGAATTTCAGTCTC	This Paper	N/A
sg*Sf3b1* forward4: CACCGATTACTATGCTAGAGTGGA	This Paper	N/A
sg*Sf3b1* reverse4: AAACTCCACTCTAGCATAGTAATC	This Paper	N/A
sg*Sf3b2* forward1: CACCGAAGTACCTTCAAGGCAAACG	This Paper	N/A
sg*Sf3b2* reverse1: AAACCGTTTGCCTTGAAGGTACTTC	This Paper	N/A
sg*Sf3b2* forward2: CACCGTCGTCCTATAGGGAGTCGCG	This Paper	N/A
sg*Sf3b2* reverse2: AAACCGCGACTCCCTATAGGACGAC	This Paper	N/A
sg*Sf3b3* forward1: CACCGAACACTCTGGTGTATCACG	This Paper	N/A
sg*Sf3b3* reverse1: AAACCGTGATACACCAGAGTGTTC	This Paper	N/A
sg*Sf3b3* forward2: CACCGATGGCGTTTAGGCTAACAGG	This Paper	N/A
sg*Sf3b3* reverse2: AAACCCTGTTAGCCTAAACGCCATC	This Paper	N/A
sg*Sf3b4* forward1: CACCGGTCAACACCCACATGCCCA	This Paper	N/A
sg*Sf3b4* reverse1: AAACTGGGCATGTGGGTGTTGACC	This Paper	N/A
sg*Sf3b4* forward2: CACCGAACTCCAAGCTGTACCTGG	This Paper	N/A
sg*Sf3b4* reverse2: AAACCCAGGTACAGCTTGGAGTTC	This Paper	N/A
sg*Sf3b5* forward1: CACCGCCACGCCGACACCACCAAG	This Paper	N/A
sg*Sf3b5* reverse1: AAACCTTGGTGGTGTCGGCGTGGC	This Paper	N/A
sg*Sf3b5* forward2: CACCGACTCCTACTGCTCCTACAT	This Paper	N/A
sg*Sf3b5* reverse2: AAACATGTAGGAGCAGTAGGAGTC	This Paper	N/A
sg*Sf3b14* forward1: CACCGAAACGTTGAATCCTGATAGG	This Paper	N/A
sg*Sf3b14* reverse1: AAACCCTATCAGGATTCAACGTTTC	This Paper	N/A
sg*Sf3b14* forward2: CACCGAATCCTGATAGGTGGTCGC	This Paper	N/A
sg*Sf3b14* reverse2: AAACGCGACCACCTATCAGGATTC	This Paper	N/A
sg*Phf5a* forward1: CACCGCAGGGTGCAGGGACGCACGT	This Paper	N/A
sg*Phf5a* reverse1: AAACACGTGCGTCCCTGCACCCTGC	This Paper	N/A
sg*Phf5a* forward2: CACCGCTCATCACATATGCGGACCA	This Paper	N/A
sg*Phf5a* reverse2: AAACTGGTCCGCATATGTGATGAGC	This Paper	N/A
sg*Zbp1* forward1: CACCGTGAGCTATGACGGACAGACG	This Paper	N/A
sg*Zbp1* reverse1: AAACCGTCTGTCCGTCATAGCTCAC	This Paper	N/A
sg*Zbp1* forward2: CACCGCTTCCCTGCATCCCCCACG	This Paper	N/A
sg*Zbp1* reverse2: AAACCGTGGGGGATGCAGGGAAGC	This Paper	N/A
sg*Zbp1* forward3: CACCGCAGGTGTTGAGCGATGACGG	This Paper	N/A
sg*Zbp1* reverse3: AAACCCGTCATCGCTCAACACCTGC	This Paper	N/A
sg*Zbp1* forward4: CACCGTGTGCTGACAAATAATCGCA	This Paper	N/A
sg*Zbp1* reverse4: AAACTGCGATTATTTGTCAGCACAC	This Paper	N/A
*mArc* forward: CACAGGAAGCAGCAAGATGGTG	This Paper	N/A
*mArc* reverse: CCTATCCTGACCAAGCCTCAG	This Paper	N/A
*mCactin* forward: GGCTTTCACAGGGCCTTGGCA	This Paper	N/A
*mCactin* reverse: AGGAGGGTGTCACTATCTCAGTC	This Paper	N/A
*mChpf* forward: CAGGGGAAAAAGGGGCCATGAGC	This Paper	N/A
*mChpf* reverse: CTGGGCTTAGGGAGTCAAGGGA	This Paper	N/A
*mCluh* forward: CCTCTGCCACCATGCCCTGC	This Paper	N/A
*mCluh* reverse: TGTGGCAGGCAGGGGAATGGA	This Paper	N/A
*mPcyt2* forward: CTAGAGGTGACACGCACACAGAA	This Paper	N/A
*mPcyt2* reverse: CAAACCCTGTTTATGAGCCTAGTG	This Paper	N/A
*mActb* forward: GGCTGTATTCCCCTCCATCG	This Paper	N/A
*mActb* reverse: CCAGTTGGTAACAATGCCATGT	This Paper	N/A
*mGapdh* forward: TGACCTCAACTACATGGTCTACA	This Paper	N/A
*mGapdh* reverse: CTTCCCATTCTCGGCCTTG	This Paper	N/A
Recombinant DNA		
lentiCRISPR v2	Addgene	Cat# 52961; RRID: Addgene_52961
pMD2.G	Addgene	Cat# 12259; RRID: Addgene_12259
pMD2.G	Addgene	Cat# 12260; RRID: Addgene_12260
Plasmid: sg*SF3B1 #3*	This Paper	N/A
Plasmid: sg*SF3B1 #4*	This Paper	N/A
Plasmid: sg*Sf3b1 #3*	This Paper	N/A
Plasmid: sg*Sf3b1 #3*	This Paper	N/A
Plasmid: sg*Sf3b2 #1*	This Paper	N/A
Plasmid: sg*Sf3b2 #2*	This Paper	N/A
Plasmid: sg*Sf3b3 #1*	This Paper	N/A
Plasmid: sg*Sf3b3 #2*	This Paper	N/A
Plasmid: sg*Sf3b4 #1*	This Paper	N/A
Plasmid: sg*Sf3b4 #2*	This Paper	N/A
Plasmid: sg*Sf3b5 #1*	This Paper	N/A
Plasmid: sg*Sf3b5 #2*	This Paper	N/A
Plasmid: sg*Sf3b14 #1*	This Paper	N/A
Plasmid: sg*Sf3b14 #2*	This Paper	N/A
Plasmid: sg*Phf5a #1*	This Paper	N/A
Plasmid: sg*Phf5a #2*	This Paper	N/A
Plasmid: sg*Zbp1 #1*	This Paper	N/A
Plasmid: sg*Zbp1 #2*	This Paper	N/A
Plasmid: sg*Zbp1 #3*	This Paper	N/A
Plasmid: sg*Zbp1 #4*	This Paper	N/A
Software and algorithms		
GraphPad Prism	Dotmatics	https://www.graphpad.com/features
ImageJ	NIH	https://www.imagej.net
Biorender	Biorender	https://www.biorender.com
R	The R Project for Statistical Computing	https://www.r-project.org
FlowJo	BD Sciences	https://flowjo.com/solutions/flowjo
IGV: Integrative Genomics Viewer	Broad Institute	https://www.igv.org
Morpheus	Broad Institute	https://www.software.broadinstitute.org/morpheus
CRISPick	Broad Institute	https://portals.broadinstitute.org/gppx/crispick/public.
RNAfold	RNAfold Web Server	http://rna.tbi.univie.ac.at/cgi-bin/RNAWebSuite/RNAfold.cgi
Nextflow nf-core/RNAseq pipeline	Ewels et al.^82^	https://nf-co.re/rnaseq/3.14.0/
FASTQC	Brown et al.^83^	https://www.bioinformatics.babraham.ac.uk/projects/fastqc/
STAR Aligner	Dobin et al.^84^	https://github.com/alexdobin/STAR
Salmon	Patro et al.^85^	https://combine-lab.github.io/salmon/
DESeq2	Love et al.^86^	https://bioconductor.org/packages/devel/bioc/vignettes/DESeq2/inst/doc/DESeq2.html
iREAD (version 0.8.9).	Li et al.^87^	https://github.com/genemine/iread
Limma package	Robert et al.^88^	https://bioconductor.org/packages/release/bioc/html/limma.html
eBayes (edgeR package)	Robinson et al.^89^	https://www.bioconductor.org/packages//2.7/bioc/vignettes/edgeR/inst/doc/edgeR.pdf
Incucyte Software	Sartorius	https://downloads.essenbioscience.com/get/incucyte-2024b-gui

## Highlights

- Spliceosome inhibition induces Z-RNA accumulation
- Spliceosome inhibition triggers ZBP1-mediated death in necroptosis-competent cells
- Spliceosome inhibition induces ZBP1-mediated antitumor immunity in SCLC mouse model
- ZBP1 is essential for spliceosome inhibition to enhance ICB responses in SCLC model
