# Supplementary material for: Spliceosome inhibition induces Z-RNA and ZBP1-driven cell death in small cell lung cancer
Source: Cell Rep. Author manuscript; Available in PMC 2026 Feb 16. (PMC12908447; doi:10.1016/j.celrep.2025.116384)
Supplement: Supplementary Figures (Figures S1-S7) [file NIHMS2143456-supplement-Supplementary_Figures__Figures_S1-S7_.pdf]

Cell Reports, Volume *44*

Supplemental information

Spliceosome inhibition induces Z-RNA and ZBP1-driven cell death in small cell lung cancer

Xinpei Jiang, Xueying Ma, Yunyun Zhou, Xiaodan Liu, Ting Zhang, William Kim, Siddharth Balachandran, and Israel Cañadas

SUPPLEMENTAL INFORMATION

Figure S1

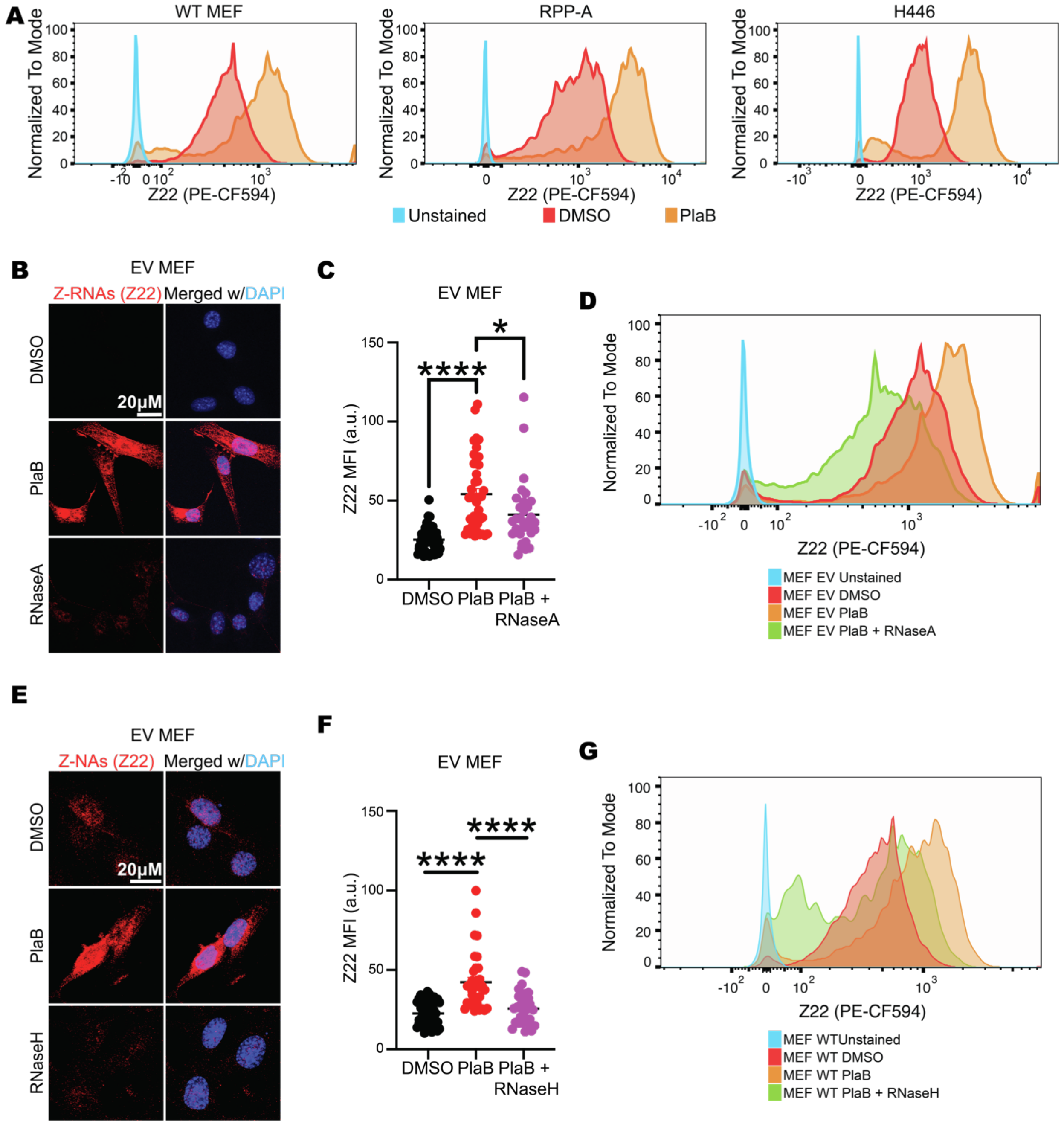

Figure S1. Pharmacological spliceosome inhibition triggers accumulation of cellular Z-RNA.

(A) Intracellular Z-RNA levels in WT MEFs, RPP-A, and H446 cells following 50nM of PlaB treatment after 18 hours were analyzed by flow cytometry.
(B-D) Spliceosome inhibition leads to the accumulation of cellular Z-RNA in EV MEFs. (B) Immunofluorescence (IF) images of Z-RNA (red) and DAPI (blue) in EV MEF cells treated with 50nM PlaB for 18 hours ± RNase A. Scale bar, 20 μM. (C) Mean fluorescence intensity (MFI) quantification in arbitrary unit (a.u.) in EV MEF cells from experiment indicated in (B). (D) Intracellular Z-RNA levels in EV MEF cells ± 50nM PlaB at 18 hours were analyzed by flow cytometry ± RNase A treatment.
(E-G) RNase H treatment reduces the Z22 signal induced by spliceosome inhibition. (E) IF staining images of Z-RNA (red) and DAPI (blue) in EV MEF cells treated with 50nM PlaB for 18 hours ± RNase H treatment. Scale bar, 20 μM. (F) MFI quantification in arbitrary unit in EV MEF cells from experiment indicated in (E). (G) Intracellular Z-NAs levels in WT MEF cells ± 50nM PlaB at 18 hours were analyzed by flow cytometry ± RNase H treatment.
All quantification plots of Z-RNA signal intensity are mean ± SEM, two-tailed unpaired Student’s test. *$p < 0.05$, ****$p < 0.0001$. Representative data from at least three independent experiments are shown.

Figure S2

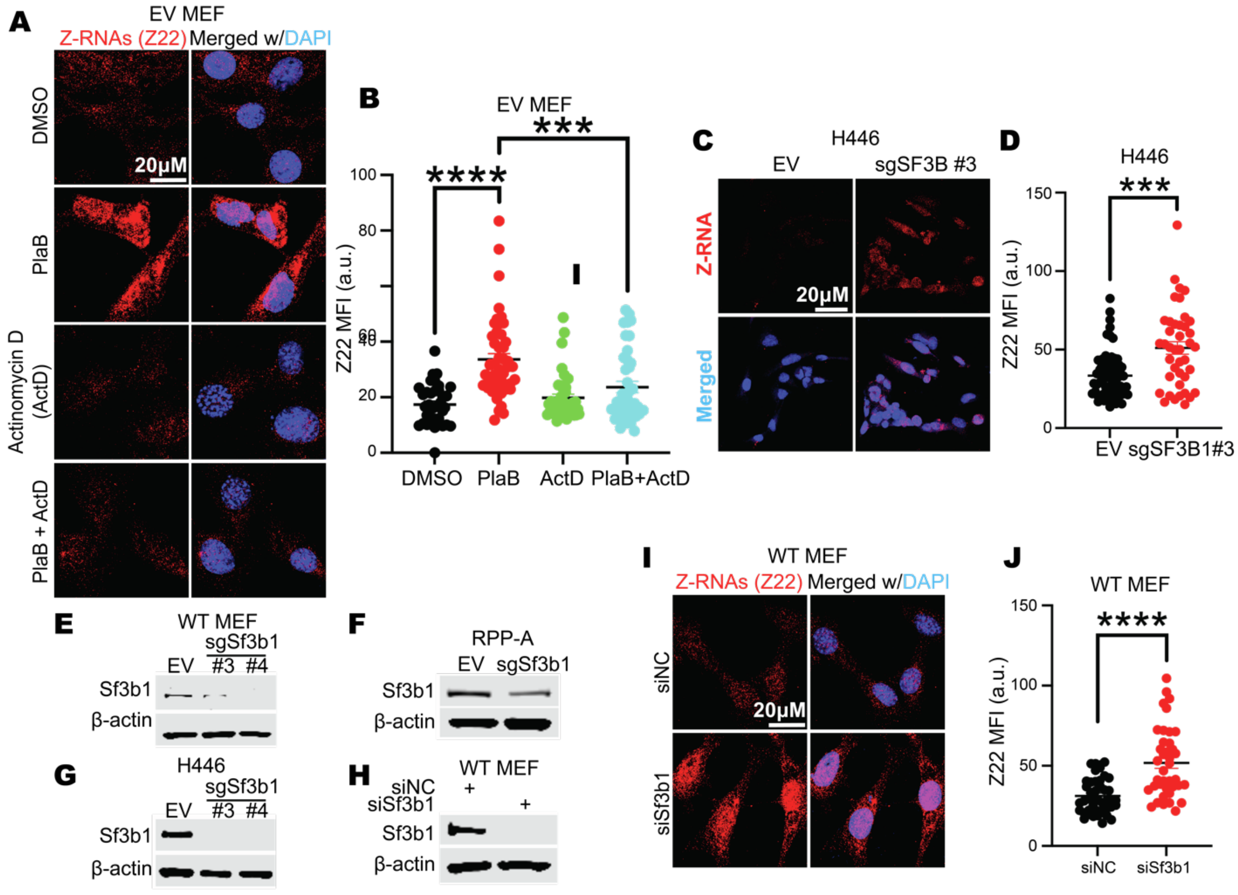

A
EV MEF
Z-RNAs (Z22)
Merged w/DAPI
DMSO
20μM
PlaB
Actinomycin D (ActD)
PlaB + ActD
B
EV MEF
****
***
Z22 MFI (a.u.)
100
80
60
40
20
0
DMSO
PlaB
ActD
PlaB+ActD
C
H446
EV
sgSF3B #3
Z-RNA
20μM
Merged
D
H446
***
Z22 MFI (a.u.)
150
100
50
0
EV
sgSF3B1#3
E
WT MEF
sgSf3b1
EV
#3
#4
Sf3b1
β-actin
F
RPP-A
EV
sgSf3b1
Sf3b1
β-actin
G
H446
sgSf3b1
EV
#3
#4
Sf3b1
β-actin
H
WT MEF
siNC
+
siSf3b1
+
Sf3b1
β-actin
I
WT MEF
Z-RNAs (Z22)
Merged w/DAPI
siNC
20μM
siSf3b1
J
WT MEF
****
Z22 MFI (a.u.)
150
100
50
0
siNC
siSf3b1

Figure S2. Genetic depletion of SF3B1 induces cellular Z-RNA accumulation.

(A and B) 1 hour of actinomycin D (ActD) pretreatment completely diminished the cellular Z-RNA signal induced by 50nM PlaB treament. (A) IF staining of Z-RNA (red) and DAPI (blue) in EV MEF cells ± 50nM PlaB at 18 hours ± 1 hour ActD pretreatment. Scale bar, 20 μM. (B) MFI quantification in EV MEF cells from experiment indicated in (A).

(C and D) Depletion of SF3B1 by CRISPR-Cas9 method induced the accumulation of cellular Z-RNA. (C) IF staining images of Z-RNA (red) and DAPI (blue) of H446 cells infected with EV or sgSF3B1. Scale bar, 20 μM. (D) MFI quantification in H446 cells from experiment indicated in (C).

(E-G) Immunoblots of SF3B1 and β-actin in WT MEF (E), RPP-A (F), and H446 (G) cells infected with EV or sgSF3B1. CRISPR-Cas9 mediated knockdown experiments were performed using pooled cell populations after antibiotic selection.

(H-J) siRNA knockdown of Sf3b1 recapitulated the pharmacological inhibition and genetic depletion of SF3B1. (H) Immunoblots of SF3B1 and β-actin in WT MEF cells transfected with siRNA non-targeting control (siNC) or siSf3b1, and (I) IF staining images of Z-RNA (red) and DAPI (blue) of WT MEF cells transfected with siNC or siSf3b1. Scale bar, 20 μM. (J) MFI quantification for H446 cells from experiment indicated in (I).

All quantification plots of Z-RNA signal intensity are mean ± SEM, two-tailed unpaired Student’s test. *$p < 0.05$, ***$p < 0.001$, ****$p < 0.0001$. Representative data from at least three independent experiments are shown.

Figure S3

A

RNA	Count
non-mRNA	44
pre-mRNA	970

B

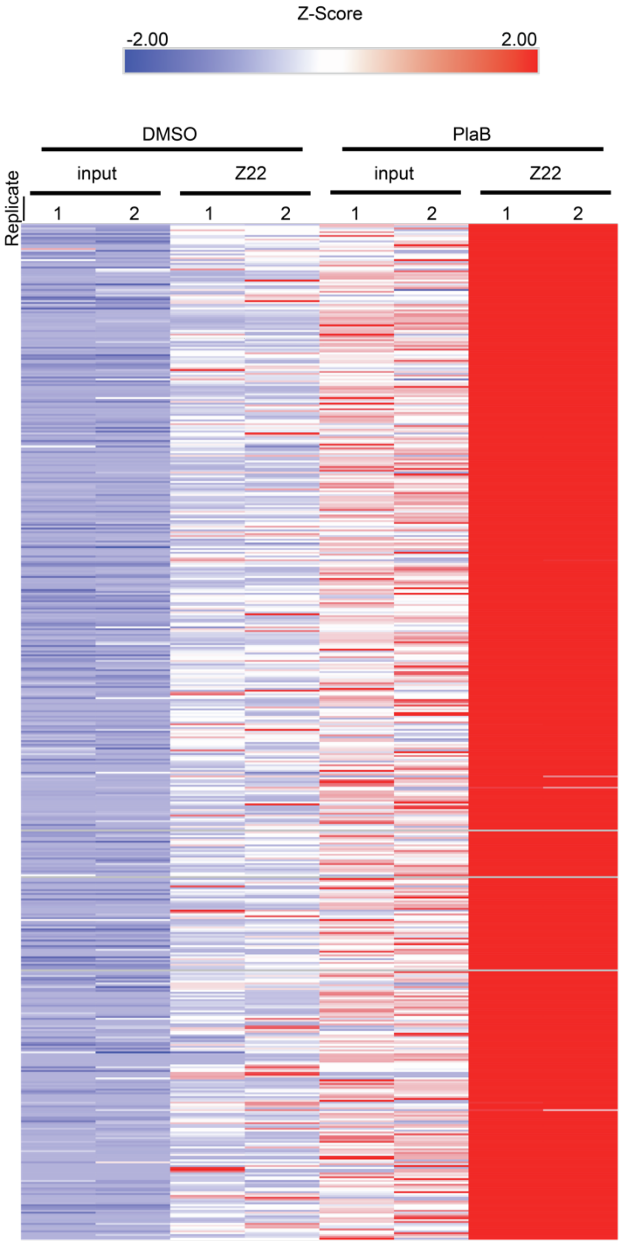

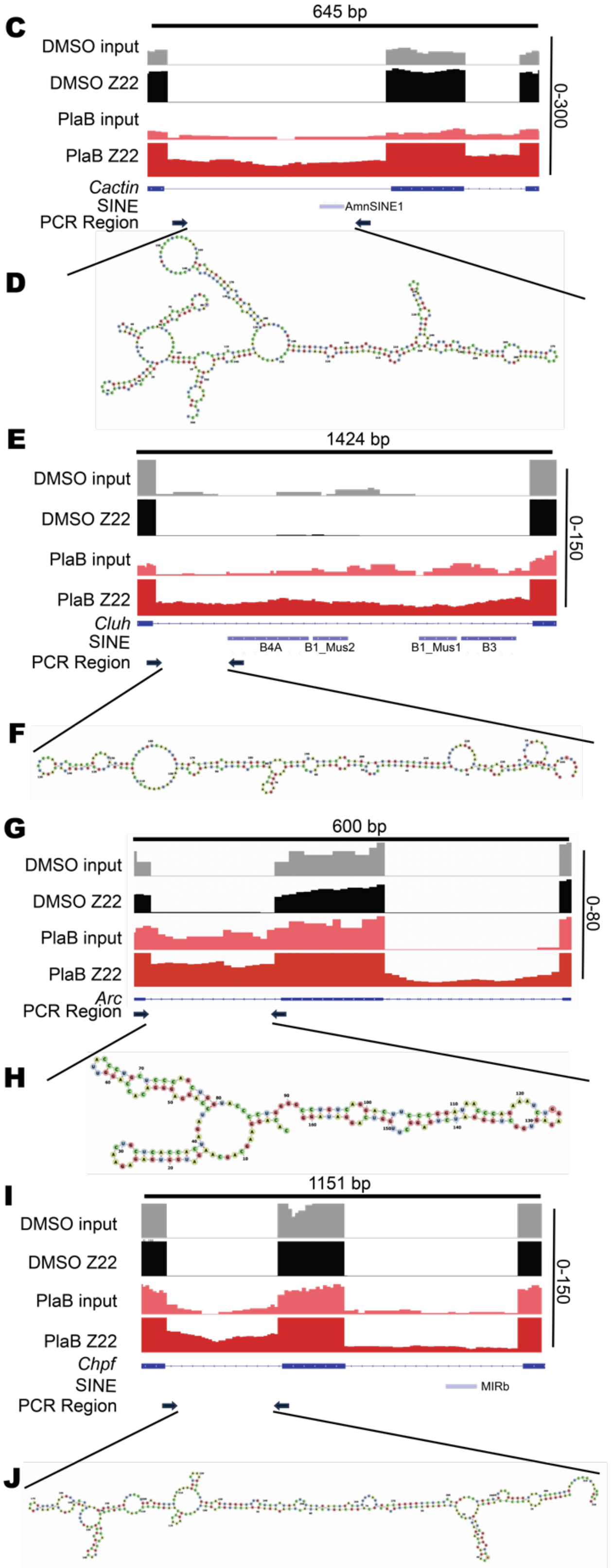

Figure S3. Spliceosome inhibition leads to pre-mRNA intron retention with high propensity to form ds-Z-RNA secondary structures.

(A) Frequency counts of Z22 immunoprecipitation PlaB enriched RNA species.

(B) Heatmap of z-score normalized FPKM for all significantly enriched retained intron RNA species from RIP-Seq analysis in EV MEFs treated with PlaB vs DMSO. Data represent two independent biological replicates.

(C-F) Retained introns with retrotransposons induced by 50nM PlaB can form double stranded (ds)-Z-RNA secondary structures. (C) *Cactin* gene Z-RNA intronic signals visualized by Integrative Genomics Viewer (IGV), and (D) Z22-enriched retrotransposon containing intron in the *Cactin gene* predicted to form dsRNA secondary structure. (E) IGV image displaying the Z22 intronic enrichment in *Cluh* gene and (F) *Cluh* retained intron predicted to form dsRNA secondary structure.

(G-J) Introns not containing retrotransposons can also form double stranded secondary structures. (G) IGV visualization of introns in *Arc*, and (H) predicted double-stranded secondary structure. (I) IGV image depicting the introns and exons in *Chpf* gene, and (J) predicted dsRNA secondary structure.

Figure S4

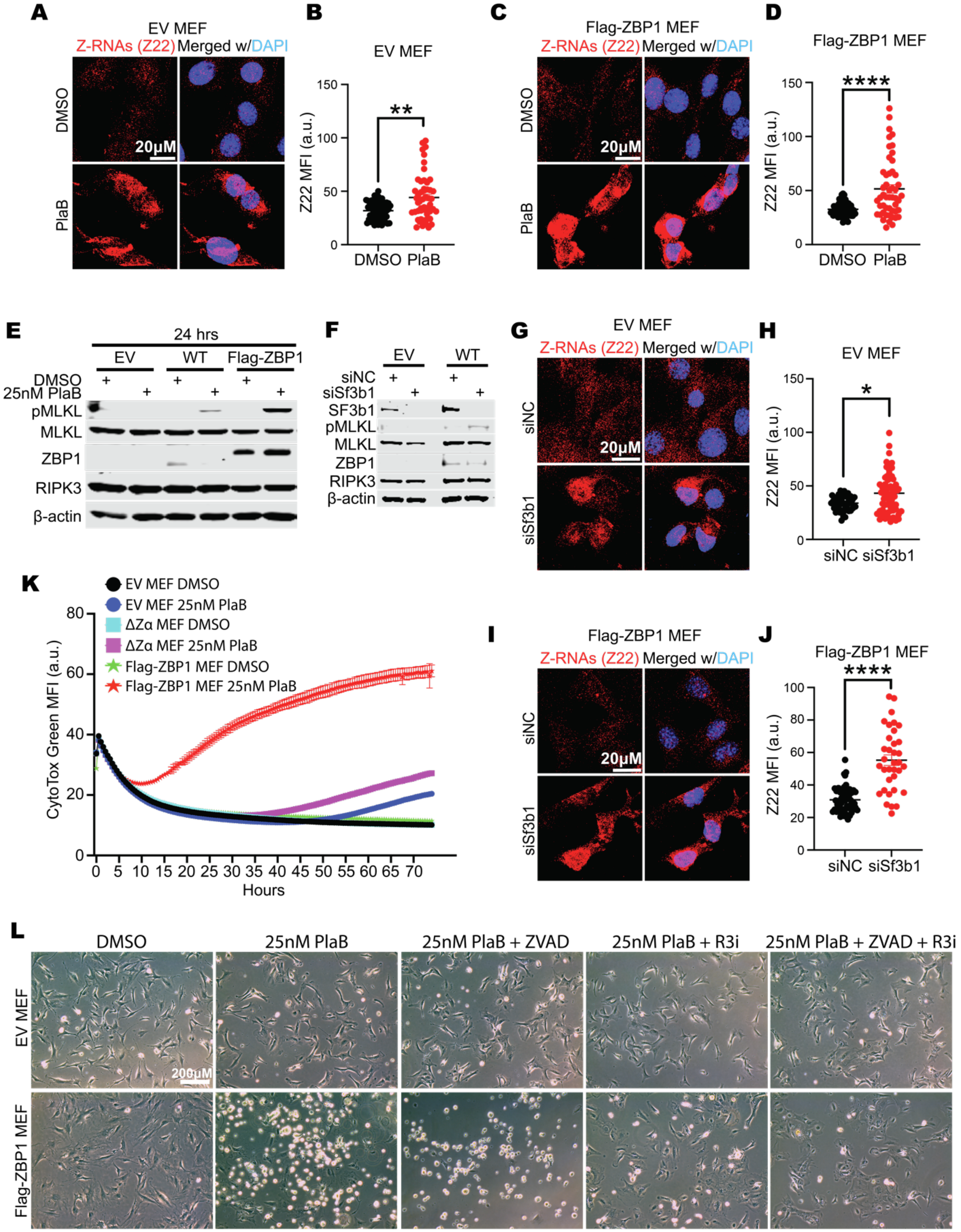
A
EV MEF
Z-RNAs (Z22) Merged w/DAPI
DMSO
PlaB
20μM
B
EV MEF
Z22 MFI (a.u.)
**
DMSO PlaB
C
Flag-ZBP1 MEF
Z-RNAs (Z22) Merged w/DAPI
DMSO
PlaB
20μM
D
Flag-ZBP1 MEF
Z22 MFI (a.u.)
****
DMSO PlaB
E
24 hrs
EV WT Flag-ZBP1
DMSO
25nM PlaB
pMLKL
MLKL
ZBP1
RIPK3
β-actin
F
EV WT
siNC
siSf3b1
SF3b1
pMLKL
MLKL
ZBP1
RIPK3
β-actin
G
EV MEF
Z-RNAs (Z22) Merged w/DAPI
siNC
siSf3b1
20μM
H
EV MEF
Z22 MFI (a.u.)
*
siNC siSf3b1
K
EV MEF DMSO
EV MEF 25nM PlaB
ΔZα MEF DMSO
ΔZα MEF 25nM PlaB
Flag-ZBP1 MEF DMSO
Flag-ZBP1 MEF 25nM PlaB
CytoTox Green MFI (a.u.)
Hours
I
Flag-ZBP1 MEF
Z-RNAs (Z22) Merged w/DAPI
siNC
siSf3b1
20μM
J
Flag-ZBP1 MEF
Z22 MFI (a.u.)
****
siNC siSf3b1
L
DMSO
25nM PlaB
25nM PlaB + ZVAD
25nM PlaB + R3i
25nM PlaB + ZVAD + R3i
EV MEF
Flag-ZBP1 MEF
200μM

Figure S4. SF3B1 inhibition and silencing induce cellular Z-RNA accumulation leading to ZBP1-dependent cell death that is rescued by inhibiting RIPK3 activity.

(A-D) IF images of intracellular Z-RNA levels in EV MEF cells (A) and Flag-ZBP1 MEF cells (C) treated with 50nM PlaB for 18 hours. Z-RNA (red) and DAPI (blue). Scale bar, 20 μM. MFI quantification in arbitrary unit (a.u.) in EV MEF cells (B) from experiment indicated in (A), and Flag-ZBP1 MEF Cells (D) from experiment indicated in (C).

(E) Immunoblots of MLKL activation (pMLKL) in EV MEFs, WT MEFs, and Flag-ZBP1 MEFs treated with PlaB after 18 hours.

(F) Immunoblots of MLKL activation (pMLKL) in EV MEFs and WT MEFs after siNC and siSf3b1 knockdown at 72 hours.

(G-J) Immunofluorescence staining images of Z-RNA (red) and DAPI (blue) in EV MEF cells (G) and Flag-ZBP1 MEF cells (I) transfected with siNC or siSf3b1. Scale bar, 20 μM. Mean fluorescence intensity quantification for EV MEF cells (H) from experiment indicated in (G), and Flag-ZBP1 MEF (J) cells from experiment indicated in (I).

(K) 72 hours Real-time Incucyte Cytotoxicty Assay for Live and Dead analysis in EV MEF, ΔZα mutant MEF, and Flag-ZBP1 MEF cells treated with PlaB.

(L) Phase contrast bright field images of EV MEFs and Flag-ZBP1 MEFs treated for 18 hours with PlaB, Z-Vad (50μM), or RIPK3 inhibitor (R3i, GSK843, 5μM).

All quantification plots of Z-RNA signal intensity are mean ± SEM, two-tailed unpaired Student's test. $*p < 0.05$, $****p < 0.0001$. Data are from at least three independent experiments.

Figure S5

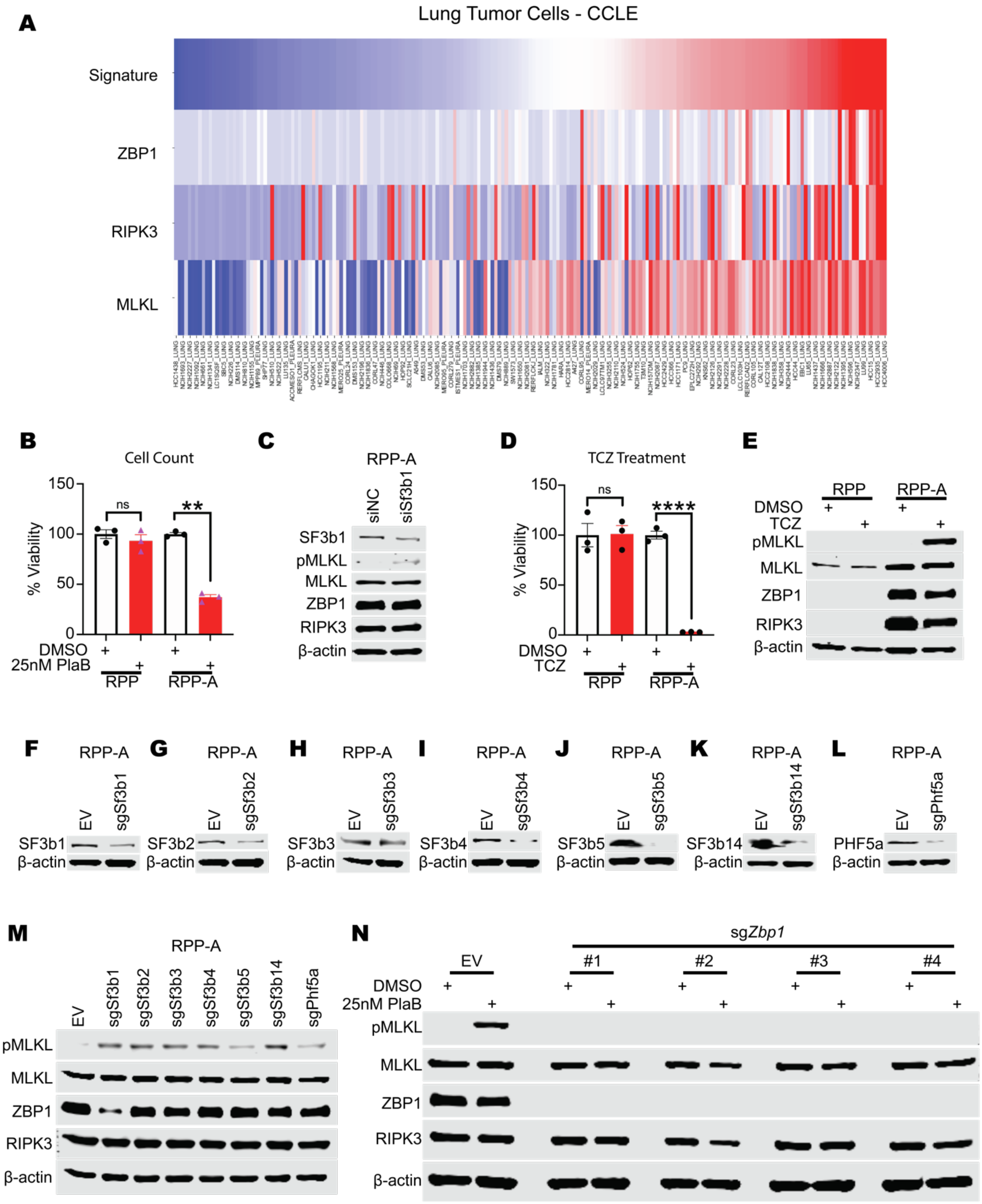
A
Lung Tumor Cells - CCLE
Signature
ZBP1
RIPK3
MLKL
B
Cell Count
% Viability
150
100
50
0
ns
**
DMSO
25nM PlaB
RPP
RPP-A
C
RPP-A
siNC
siSf3b1
SF3b1
pMLKL
MLKL
ZBP1
RIPK3
β-actin
D
TCZ Treatment
% Viability
ns
****
DMSO
TCZ
RPP
RPP-A
E
RPP
RPP-A
DMSO
TCZ
pMLKL
MLKL
ZBP1
RIPK3
β-actin
F
RPP-A
EV
sgSf3b1
SF3b1
β-actin
G
RPP-A
EV
sgSf3b2
SF3b2
β-actin
H
RPP-A
EV
sgSf3b3
SF3b3
β-actin
I
RPP-A
EV
sgSf3b4
SF3b4
β-actin
J
RPP-A
EV
sgSf3b5
SF3b5
β-actin
K
RPP-A
EV
sgSf3b14
SF3b14
β-actin
L
RPP-A
EV
sgPhf5a
PHF5a
β-actin
M
RPP-A
EV
sgSf3b1
sgSf3b2
sgSf3b3
sgSf3b4
sgSf3b5
sgSf3b14
sgPhf5a
pMLKL
MLKL
ZBP1
RIPK3
β-actin
N
sgZbp1
EV
#1
#2
#3
#4
DMSO
25nM PlaB
pMLKL
MLKL
ZBP1
RIPK3
β-actin

Figure S5. Spliceosome inhibition induces ZBP1-dependent cell death in necroptosis competent SCLC cells and rescued by ZBP1 deletion.

(A) Heatmap of necroptosis signature genes, *ZBP1*, *MLKL*, and *RIPK3* in lung cancer cell lines from the Cancer Cell Line Encyclopedia (CCLE).
(B) RPP (necroptosis incompetent) and RPP-A (necroptosis competent) cells viability after 18 hours of PlaB treatment.
(C) Western immunoblots of MLKL activation (pMLKL) in RPP-A cells after Sf3b1 knock-down.
(D) Viability in RPP and RPP-A cells after 18 hours of treatment with necroptosis inducing TCZ cocktail (Tumor Necrosis Factor combined with cycloheximide and the caspase inhibitor zVAD-fmk).
(E) Immunoblots of MLKL activation (pMLKL) in RPP-A after 18 hours of treatment with TCZ cocktail.
(F-M) Dysfunctional Sf3b complex leads to necroptosis induction in RPP-A cells. Immunoblots of CRISPR-Cas9 mediated knockdown of (F) Sf3b1, (G) Sf3b2, (H) Sf3b3, (I) Sf3b4, (J) Sf3b5, (K) Sf3b14, and (L) Phf5a in RPP-A cell line. (M) Western immunoblots of pMLKL induction after knockdown of the core components of the Sf3b complex. CRISPR-Cas9 mediated knockdown experiments were performed using pooled cell populations after antibiotic selection.
(N) Western Immunoblots of parental RPP-A cells (+ EV) and RPP-A single cell clones lacking ZBP1 (*sgZbp1) at* 18 hours of PlaB treatment.
All viability graphs are mean ± SEM, two-tailed unpaired Student's test. Not significant (ns), $**p < 0.01$, $****p < 0.0001$.

Figure S6

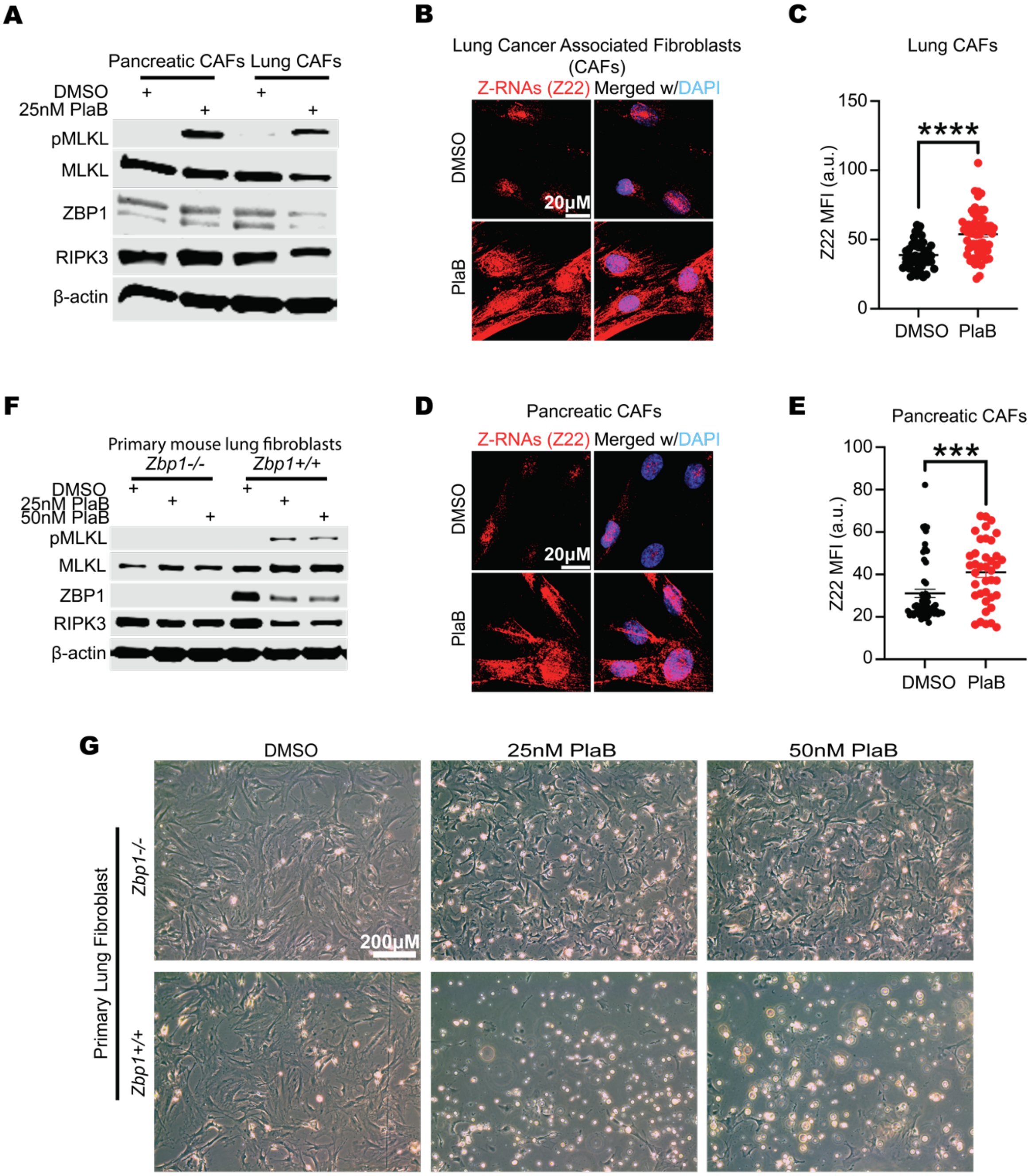
A
Pancreatic CAFs Lung CAFs
DMSO
25nM PlaB
pMLKL
MLKL
ZBP1
RIPK3
β-actin
B
Lung Cancer Associated Fibroblasts (CAFs)
Z-RNAs (Z22) Merged w/DAPI
DMSO
PlaB
20µM
C
Lung CAFs
Z22 MFI (a.u.)
150
100
50
0
****
DMSO PlaB
F
Primary mouse lung fibroblasts
Zbp1-/- Zbp1+/+
DMSO
25nM PlaB
50nM PlaB
pMLKL
MLKL
ZBP1
RIPK3
β-actin
D
Pancreatic CAFs
Z-RNAs (Z22) Merged w/DAPI
DMSO
PlaB
20µM
E
Pancreatic CAFs
Z22 MFI (a.u.)
100
80
60
40
20
0
***
DMSO PlaB
G
DMSO
25nM PlaB
50nM PlaB
Primary Lung Fibroblast
Zbp1-/-
Zbp1+/+
200µM

Figure S6. Spliceosome inhibition in CAFs triggers Z-RNA accumulation and ZBP1-dependent cell death.

(A) Western immunoblots of MLKL activation (pMLKL) in pancreatic and lung CAFs treated with PlaB.

(B-E) Spliceosome inhibition leads to the accumulation of cellular Z-RNA in human CAFs. Immunofluorescence staining images of Z-RNA (red) and DAPI (blue) in lung CAFs (B) and pancreatic CAFs (D) treated with 50nM PlaB for 18 hours. Mean fluorescence intensity quantification in arbitrary unit (a.u.) in lung CAFs (C) and pancreatic CAFs (E). Scale bar, 20 μM.

(F) Immunoblots of PlaB treated primary mouse lung fibroblasts isolated from *Zbp1*$^{-/-}$ and *Zbp1*$^{+/+}$ mice from C57BL/6J genetic background.

(G) Phase contrast bright field images displaying primary mouse lung fibroblasts after 18 hours of PlaB treatments. Scale bar, 200 μM.

All quantifications are mean ± SEM, two-tailed unpaired Student's test. ***p < 0.001, and ****p < 0.0001.

Figure S7

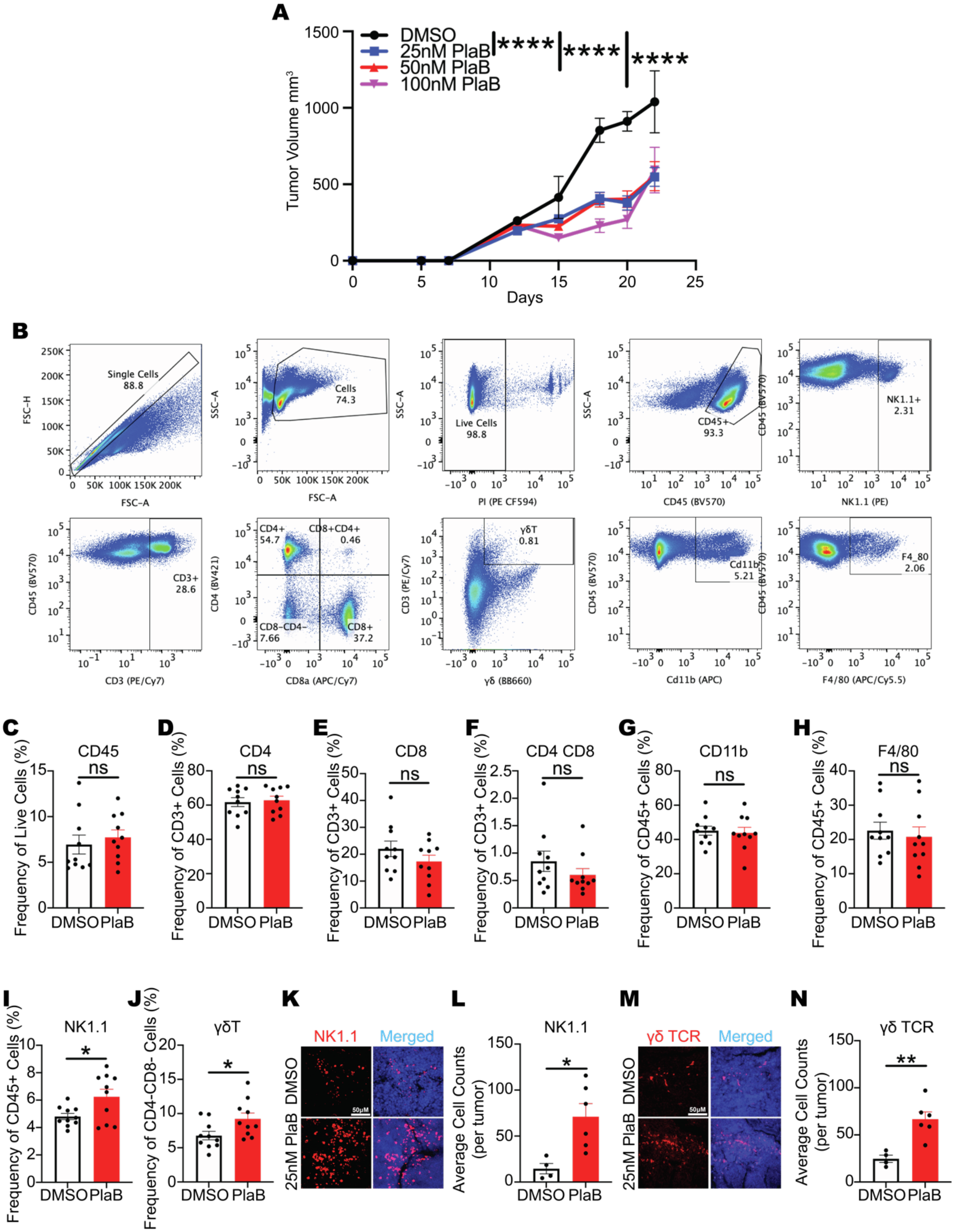

A
DMSO
25nM PlaB
50nM PlaB
100nM PlaB
****
Tumor Volume mm3
Days
B
Single Cells 88.8
Cells 74.3
Live Cells 98.8
CD45+ 93.3
NK1.1+ 2.31
CD3+ 28.6
CD4+ 54.7
CD8+CD4+ 0.46
CD8−CD4− 7.66
CD8+ 37.2
γδT 0.81
Cd11b 5.21
F4_80 2.06
FSC-A
FSC-H
SSC-A
PI (PE CF594)
CD45 (BV570)
NK1.1 (PE)
CD3 (PE/Cy7)
CD4 (BV421)
CD8a (APC/Cy7)
γδ (BB660)
Cd11b (APC)
F4/80 (APC/Cy5.5)
C
CD45
Frequency of Live Cells (%)
D
CD4
Frequency of CD3+ Cells (%)
E
CD8
F
CD4 CD8
G
CD11b
Frequency of CD45+ Cells (%)
H
F4/80
ns
DMSO
PlaB
I
NK1.1
J
γδT
Frequency of CD4-CD8- Cells (%)
K
NK1.1
Merged
DMSO
25nM PlaB
50μM
L
Average Cell Counts (per tumor)
M
γδ TCR
N
*
**

Figure S7. Intratumoral PlaB injection reduces tumor growth and increases immune infiltrates.

(A) Mouse RPP SCLC cells tumor volume measurement after subcutaneous inoculation into syngeneic C57BL/6J mice followed by intratumoral injection of DMSO or PlaB (25nM, 50nM, and 100nM) in PBS (n=6).
(B) Schematic of flow cytometry gating strategies for the immune infiltrates analysis.
(C-J) DMSO or PlaB intratumorally treated mouse tumors were digested into single cells and separated based on the immune markers (n=10). (C) CD45, (D) CD4, (E) CD8, (F) CD4 CD8, (G) CD11b, (H) F4/80, (I) NK1.1, and (J) γδT.
(K and L) Tumor sections were stained for NK1.1 immune marker. (K) Representative NK1.1 staining for tumors treated with DMSO or PlaB, and (L) quantification of NK1.1 positive cells per field per tumor (n=6).
(M and N) Tumor sections were stained for γδT immune marker. (M) Representative γδT staining for tumors treated with DMSO or PlaB, and (N) quantification of γδT positive cells per field per tumor (n=6).
P-values were calculated by Two-way ANOVA test, ****$p < 0.0001$. All quantifications are mean ± SEM, two-tailed unpaired Student’s test. Not significant (ns), *$p < 0.05$.
